# Health Problems in Adults with Prader–Willi Syndrome of Different Genetic Subtypes: Cohort Study, Meta-Analysis and Review of the Literature

**DOI:** 10.3390/jcm11144033

**Published:** 2022-07-12

**Authors:** Anna G. W. Rosenberg, Charlotte M. Wellink, Juan M. Tellez Garcia, Karlijn Pellikaan, Denise H. Van Abswoude, Kirsten Davidse, Laura J. C. M. Van Zutven, Hennie T. Brüggenwirth, James L. Resnick, Aart J. Van der Lely, Laura C. G. De Graaff

**Affiliations:** 1 Department of Internal Medicine, Division of Endocrinology, Erasmus MC, University Medical Center Rotterdam, 3015 GD Rotterdam, The Netherlands; a.rosenberg@erasmusmc.nl (A.G.W.R.); c.wellink@erasmusmc.nl (C.M.W.); j.tellezgarcia@erasmusmc.nl (J.M.T.G.); k.pellikaan@erasmusmc.nl (K.P.); d.vanabswoude@erasmusmc.nl (D.H.V.A.); k.davidse@erasmusmc.nl (K.D.); a.vanderlelij@erasmusmc.nl (A.J.V.d.L.); 2 Dutch Center of Reference for Prader–Willi Syndrome, 3015 GD Rotterdam, The Netherlands; 3 Center for Adults with Rare Genetic Syndromes, Department of Internal Medicine, Division of Endocrinology, Erasmus MC, University Medical Center Rotterdam, 3015 GD Rotterdam, The Netherlands; 4 Academic Center for Growth Disorders, Erasmus MC, University Medical Center Rotterdam, 3015 GD Rotterdam, The Netherlands; 5 Department of Clinical Genetics, Erasmus MC, University Medical Center Rotterdam, 3015 GD Rotterdam, The Netherlands; l.vanzutven@erasmusmc.nl (L.J.C.M.V.Z.); h.bruggenwirth@erasmusmc.nl (H.T.B.); 6 Department of Molecular Genetics and Microbiology, College of Medicine, University of Florida, Gainesville, FL 32611, USA; jresnick@ufl.edu; 7 ENDO-ERN, European Reference Network on Rare Endocrine Conditions

**Keywords:** Prader–Willi syndrome, genetics, genetic variation, genotype, health problems, phenotype, uniparental disomy, paternal deletion, mutism

## Abstract

Prader–Willi syndrome (PWS) is a complex, rare genetic disorder caused by a loss of expression of paternally expressed genes on chromosome 15q11.2-q13. The most common underlying genotypes are paternal deletion (DEL) and maternal uniparental disomy (mUPD). DELs can be subdivided into type 1 (DEL-1) and (smaller) type 2 deletions (DEL-2). Most research has focused on behavioral, cognitive and psychological differences between the different genotypes. However, little is known about physical health problems in relation to genetic subtypes. In this cross-sectional study, we compare physical health problems and other clinical features among adults with PWS caused by DEL (N = 65, 12 DEL-1, 27 DEL-2) and mUPD (N = 65). A meta-analysis, including our own data, showed that BMI was 2.79 kg/m^2^ higher in adults with a DEL (*p* = 0.001). There were no significant differences between DEL-1 and DEL-2. Scoliosis was more prevalent among adults with a DEL (80% vs. 58%; *p* = 0.04). Psychotic episodes were more prevalent among adults with an mUPD (44% vs. 9%; *p* < 0.001). In conclusion, there were no significant differences in physical health outcomes between the genetic subtypes, apart from scoliosis and BMI. The differences in health problems, therefore, mainly apply to the psychological domain.

## 1. Introduction

Prader–Willi syndrome (PWS) is a complex, rare genetic disorder with an incidence of 1:16.000–1:20.000 live births [1,2]. It is characterized by intellectual disability, hypotonia, sleep-related disorders and hypothalamic dysfunction [3,4], which in their turn lead to hyperphagia, abnormal pain perception, disturbed thermoregulation and pituitary hormone deficiencies [3,4,5].

**Figure 1:**
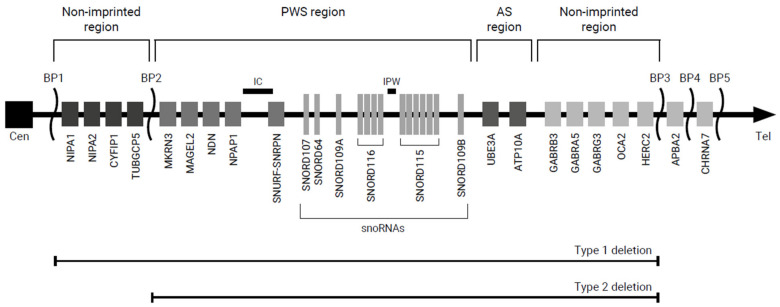
Overview of the chromosome 15q11.2-q13 region. Abbreviations: AS, Angelman syndrome; BP, breakpoint; Cen, centromere; IC, imprinting center; PWS, Prader–Willi syndrome; Tel, telomere.

PWS is caused by lack of expression of a cluster of paternally expressed genes in the PWS-region on chromosome 15q11.2-q13 [3]. The 15q11.2-q13 region can be subdivided into four distinct sub-regions: a proximal non-imprinted region, the PWS region, the Angelman syndrome (AS) region and a distal non-imprinted region (Figure 1) [3,6]. The genes in the PWS region are maternally imprinted, which means that only the paternally inherited genes are expressed. The PWS region encompasses several genes, including *MKRN3*, *MAGEL2*, *NDN*, *NPAP1*, *SNURF*-*SNRPN* and clusters of small nucleolar RNA genes (snoRNAs) (6). The AS region contains the maternally expressed gene *UBE3A* [6]. The lack of expression of this maternally expressed gene results in AS.

The primary genetic mechanisms leading to PWS are paternal deletion (DEL; 65–75%), maternal uniparental disomy (mUPD; 20–30%) and imprinting center defects (ICD; 1–3%) [6]. More rare causes of PWS are balanced translocations (0.1%) and point mutations (<0.1%) [6]. Deletions can be subdivided into type 1 and type 2 deletions (DEL-1, DEL-2). DEL-1 ranges from breakpoint (BP) 1 to BP3, which includes the proximal non-imprinted region, PWS region, AS region and distal non-imprinted region [3,6]. In DEL-2 (BP2 to BP3), the genes in the proximal non-imprinted region are normally expressed [3,6]. Around 8% of the deletions have sizes and/or locations different from DEL-1 and DEL-2 [7].

Several differences between individuals with an mUPD and DEL have been described. Individuals with a DEL tend to have more compulsive behavior [8], better jigsaw skills [9,10], a higher performance intelligence quotient (PIQ) [11] and more hypopigmentation [12,13], whereas individuals with an mUPD are reported to have milder dysmorphic features [12], more psychotic episodes [14], a higher verbal IQ (VIQ) [11,15] and more autism spectrum features [10]. There is no consensus on the differences between individuals with a DEL-1 and DEL-2. Although some studies suggest that individuals with a DEL-1 have more severe compulsive behavior [16,17] and may have delayed speech acquisition [18], other studies did not find any differences between DEL-1 and DEL-2 [19,20]. Although Coupaye et al. [21] showed that adults with a DEL might be more prone to hypothyroidism and might have a higher fat mass (FM) and lean body mass (LBM) than adults with an mUPD, most other studies only focused on differences in behavior, cognitive profile and psychiatric diagnoses and not on somatic differences. Moreover, little is known about the differences in health problems during adulthood.

In this article, we describe the clinical characteristics of 93 adults with PWS according to genotype (mUPD or DEL). Furthermore, we provide a concise overview of the current knowledge on genetic subtype differences in health problems among adults with PWS.

## 2. Materials and Methods

Approval for this study was waived by the local medical ethics committee of the Erasmus University Medical Center Rotterdam because participants were not subject to procedures or required to follow rules of behavior. In this cross-sectional study, we retrospectively reviewed the medical files of all adults with genetically confirmed PWS who visited the outpatient clinic of the Center for Adults with Complex Rare Genetic Syndromes at the Erasmus University Medical Center, Rotterdam, the Netherlands between January 2015 and June 2021. Adults with multiple genetic syndromes, such as a combination of Klinefelter and Prader–Willi syndrome, were excluded. All patients underwent a systematic health screening as part of regular patient care, including a structured interview, a medical questionnaire, a complete physical examination, biochemical measurements and a review of the medical records, as described previously [22]. The items on physical complaints, symptoms of disease and behavioral challenges were rated on a five-point Likert scale (1 = rarely or never, 2 = not often and/or not severe, 3 = quite often and/or quite severe, 4 = often and/or severe, 5 = very often and/or very severe). An item with a score of 3 or higher was considered present. Patients who had experienced at least one episode of psychotic behavior (diagnosed by a psychologist/psychiatrist) were considered as having a (history of) psychosis. Part of the clinical data of this patient group has been previously published in another context [22,23,24,25,26,27].

### 2.1. Genetic Diagnosis

Until 2018, the genetic diagnosis was made with a methylation sensitive PCR test, based on allelic methylation differences at the SNRPN locus [28]. In case of hypomethylation, fluorescent in situ hybridization (FISH) was performed with a probe for SNRPN (Prader–Willi/Angelman (SNRPN), Cytocell Ltd., Cambridge, UK) to check for the presence of a DEL as the underlying molecular cause (initially). Later, genomic dosage was assessed using multiplex ligation-dependent probe amplification (MLPA) (MLPA kit ME028, PWS/AS, MRC Holland, Amsterdam, The Netherlands). As of 2018, both methylation status and genomic dosage of the 15q11-q13 region were assessed using methylation specific MLPA.

In the absence of a DEL, the DNA of the index and the parents was tested with a set of highly polymorphic chromosome 15q markers to demonstrate the presence of an mUPD. As of 2018, this marker-test was replaced by SNP array (Infinium Global Screening Array-24 with Multi Disease add-on; Illumina, San Diego, CA, USA). If no mUPD was identified as an underlying molecular cause, we concluded that hypomethylation was caused by an imprinting defect [29].

### 2.2. Literature Search

Literature was searched in the databases Embase, MEDLINE (via ALL OVID), Web of Science Core Collection, Cochrane Central Register of Controlled Trials and Google Scholar. The full search strategy is included in the Supplementary Materials (Table S1). The literature search, completed in June 2020, only included original research articles comparing the phenotype of PWS adults with different genotypes (mUPD or DEL). We excluded reviews, conference abstracts, data from unpublished research, non-human research, articles with less than 10 subjects and non-English articles. We also excluded articles with incomplete phenotypic or genotypic data, articles describing pediatric patients (mean/median age below 18 years), articles reporting only prenatal characteristics and articles reporting genetic defects outside the chromosome 15q11.2-q13 region, including chromosome 15 duplication syndrome, trisomy 15 syndrome, AS and/or the occurrence of multiple syndromes within one individual. There were no restrictions on period. All articles were independently screened by three reviewers, first by title and abstract (CW and JTG), then by full text (CW, JTG and AR). Data were independently extracted by two reviewers (CW and AR). In case of disagreement, a fourth reviewer (LdG) was consulted.

### 2.3. Data Analysis

Data were analyzed with IBM *Statistical Package for the Social Sciences* Statistics version 25.0 and R version 3.6.0. Descriptive statistics are presented as median and interquartile range (IQR) for continuous data and as count and percentage (%) for categorical data. The chi-square test (for categorical variables) and Wilcoxon rank sum test (for continuous variables) were used to detect any differences in clinical features between the genotypes (mUPD vs. DEL and DEL-1 vs. DEL-2). Tests were only performed when at least 10 people had the genetic subtype. Logistic regression analyses were performed to detect differences in clinical features between mUPD and DEL, independent of (past or current) growth hormone (GH) treatment. Additionally, a stratified analysis for differences in health problems between the genetic subtypes was performed to visualize the impact of GH treatment. Further, a meta-analysis for BMI was performed. The result is shown as the pooled mean difference with a 95% confidence interval (CI). Data were pooled with inverse variance weighting on the number of subjects, mean and standard deviation of the studies. A fixed effect model was used when heterogeneity (assessed with *I*^2^ statistic) was <50%. *p*-values < 0.05 were considered statistically significant.

## 3. Results

A total of 111 patients (51 male and 60 female) had a genetically confirmed diagnosis of PWS. The median age was 28.5 (IQR: 21.1–38.7) years and median body mass index (BMI) was 29.1 (IQR: 26.3–37.3) kg/m^2^. Of the patients, 28 had an mUPD, 65 a DEL (12 DEL-1, 27 DEL-2) and 3 an ICD. In 15 patients, genetic material from the parents was not available, making it impossible to distinguish between an mUPD and ICD. These patients were excluded from further analyses. The patient characteristics are described in Table 1.

### 3.1. Paternal Deletion Compared to Maternal Uniparental Disomy

Gender, age and BMI did not differ significantly between adults with a DEL or mUPD (Table 1). The proportion of patients with current or past GH treatment was similar for the DEL and mUPD group. Differences in health problems, physical complaints, symptoms of disease and behavioral challenges according to genotype are described in Table 2.

Scoliosis was more often present in adults with a DEL (N = 51 out of 64, 80%) than in adults with an mUPD (N = 15 out of 26, 58%, *p* = 0.03), even after correcting for GH treatment (*p* = 0.04). Osteopenia was present in 47% of adults with a DEL (N = 21 out of 45) and 33% of adults with an mUPD (N = 7 out of 21), but this did not reach statistical significance (*p* > 0.05). Likewise, hypertension was twice as prevalent among adults with an mUPD (N = 7 out of 26, 27%) than among adults with a DEL (N = 8 out of 63, 13%), but this was not significant either. There were no differences in diagnoses of osteoporosis, epilepsy, hypercholesterolemia, diabetes mellitus type 2 (DM2) or hypothyroidism between the genotypes.

Physical complaints and behavioral challenges reported by patients and/or caregivers did not differ significantly between adults with an mUPD and DEL. In both groups, complaints of constipation and daytime sleepiness were frequent. Although not significant, abdominal pain and fatigue were present approximately three times more often in adults with a DEL. Abdominal pain was present in 17% of patients with a DEL (N = 9 out of 53) versus 5% of adults with an mUPD (N = 1 out of 22). Fatigue was present in 26% of patients with a DEL (N = 13 out of 50) versus 10% of adults with an mUPD (N = 2 out of 21). Complaints of the feet, urinary incontinence, cold intolerance and swollen legs, on the other hand, were more often reported among adults with an mUPD compared to DEL (foot complaints: 39% vs. 18%; urinary incontinence: 14% vs. 4%; cold intolerance: 30% vs. 19%; swollen legs: 30% vs. 17%). However, these differences were not statistically significant (*p* > 0.05 for all).

Twelve (44%) out of 27 adults with an mUPD had a psychotic episode compared to 6 (9%) out of 65 adults with a DEL (*p* < 0.001). Temper outbursts, food-seeking behavior and skin picking also seemed slightly more prevalent in adults with an mUPD than in adults with a DEL (temper outbursts: 43% vs. 30%; food-seeking behavior: 37% vs. 28%; skin picking: 56% vs. 42%). However, these differences were not significant. Psychotropic drugs were significantly more often used by adults with an mUPD than by adults with a DEL (*p* = 0.002; Table 1). Thirteen (50%, data missing in two adults) adults with an mUPD used psychotropic drugs, of which nine had a (history of) psychosis. Of adults with a DEL, only twelve (18%) adults used psychotropic drugs, of which four had a (history of) psychosis.

The stratified analysis on GH treatment shows that osteoporosis/osteopenia, hypercholesterolemia, DM2, obesity and hypertension rates are higher in adults who never received GH treatment (Table 3).

Among adults who were treated with GH, there were no differences in health problems between DEL and mUPD (apart from the previously mentioned scoliosis and psychotic episodes).

### 3.2. Deletion Type 1 Compared to Deletion Type 2

Gender, age and BMI did not differ significantly between adults with a DEL-1 or DEL-2 (Table 1). The proportion of patients with current or past GH treatment was similar for the DEL-1 and DEL-2 group. Differences in health problems, physical complaints, symptoms of disease and behavioral challenges between adults with a DEL-1 and DEL-2 are described in Table 4.

None of the health problems differed significantly between adults with a DEL-1 and DEL-2. Scoliosis was present in more than 80% of the adults with a DEL-1 or DEL-2. Osteopenia seemed slightly more prevalent among adults with a DEL-2 (N = 13 out of 23; 57%) compared to DEL-1 (N = 2 out of 9, 22%, *p* = 0.18). There were no differences in diagnoses of osteoporosis, psychotic episodes, epilepsy, hypercholesterolemia, DM2, hypertension and hypothyroidism between the genetic subgroups.

Physical complaints and behavioral challenges reported by the patients and/or caregivers did not differ significantly between adults with DEL-1 and DEL-2. At least 30% of the adults with a DEL-1 or DEL-2 experienced daytime sleepiness and/or temper outbursts. Although not significant, constipation and skin picking seemed more prevalent among adults with a DEL-2 compared to DEL-1 (constipation: 40% vs. 25%; skin picking: 39% vs. 20%). On the other hand, fatigue, cold intolerance, foot complaints, swollen legs and food-seeking behavior were more often reported in adults with a DEL-1 compared to DEL-2 (fatigue: 40% vs. 19%; cold intolerance: 30% vs. 14%; foot complaints: 20% vs. 5%; swollen legs: 33% vs. 10%; food-seeking behavior: 33% vs. 22%), but these differences did not reach statistical significance. There was also no significant difference in the use of psychotropic drugs between the two groups.

The stratified analysis on GH treatment shows that osteoporosis/osteopenia and obesity rates seem higher in adults who never received GH treatment (Table 5). However, there do not seem to be any differences in health problems between DEL and mUPD within the strata.

### 3.3. Literature Review

**Figure 2:**
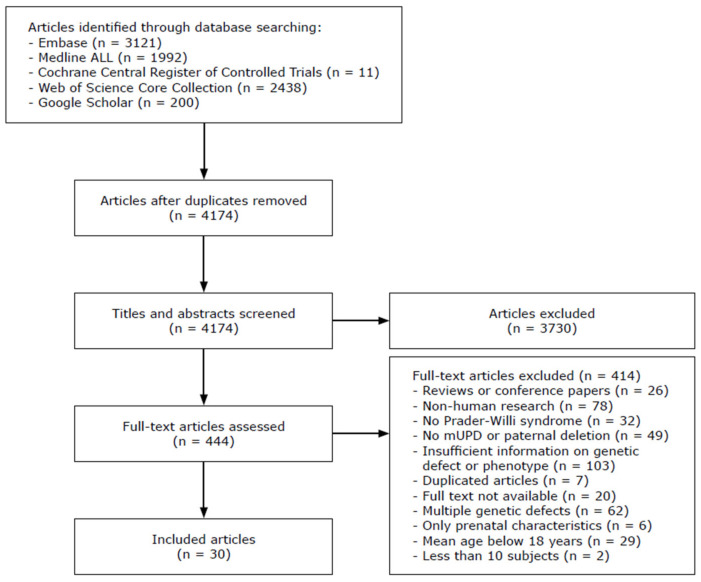
Flowchart of included studies. Abbreviations: mUPD, maternal uniparental disomy.

**Figure 3:**
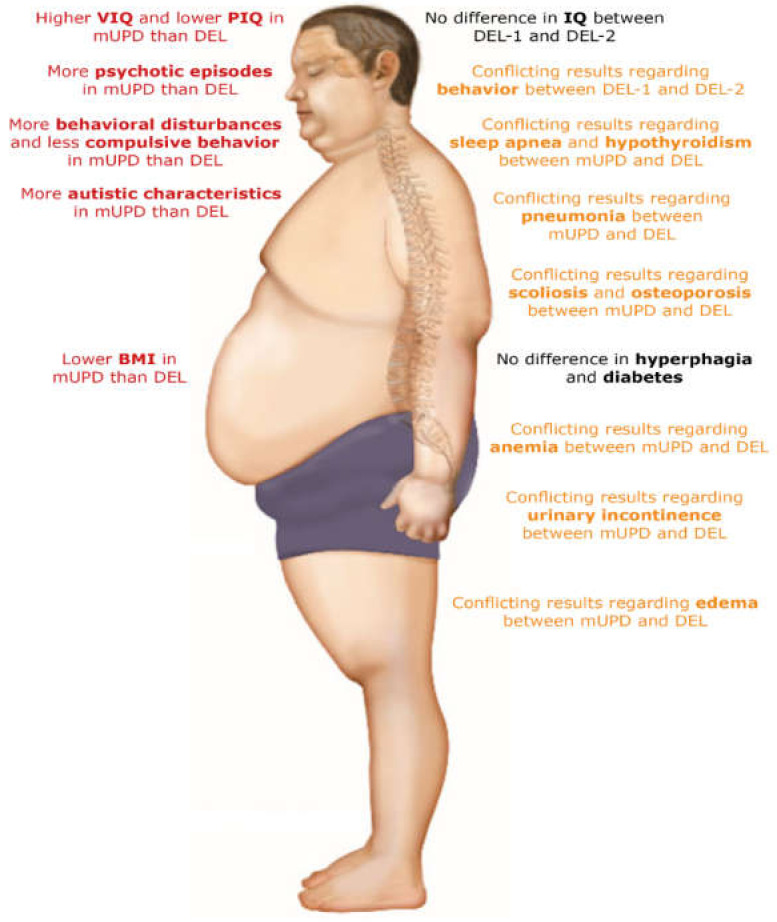
Overview of differences between adults with a maternal uniparental disomy and paternal deletion. Abbreviations: BMI, body mass index; DEL, paternal deletion; DEL-1, paternal deletion type 1; DEL-2, paternal deletion type 2; IQ, intelligence quotient; mUPD, maternal uniparental disomy; PIQ, performance intelligence quotient; VIQ, verbal intelligence quotient. The figure was adapted from Zorgatlas Groeihormoon, Esculaap Media bv, 2017 [53]. Red represents differences between groups, orange represents conflicting results regarding differences in health problems between groups and black represents no differences between groups.

The original search yielded 7762 articles. After deduplication, the titles and abstracts of 4174 articles were screened. Of 444 articles, we assessed the full text article, after which we included 30 articles in the literature review (Figure 2). The study characteristics and findings of the included articles are described in Table 6 and Table 7 and visualized in Figure 3. Most studies focused on behavior (10 studies), cognition (9 studies), psychopathology (5 studies) and/or hyperphagia (6 studies). Three studies investigated general somatic health problems [21,30,31].

There was no difference in hyperphagia between adults with an mUPD and DEL [19,21,34,37,43], nor between DEL-1 and DEL-2 [19,36]. There was no difference in the presence of scoliosis and diabetes, but hypothyroidism, sleep apnea, osteoporosis and edema might be more prevalent in patients with a DEL than in mUPD patients [21,30,31]. Moreover, FM and LBM were higher in adults with a DEL [21]. Pneumonia, anemia and urinary incontinence might be more prevalent among patients with an mUPD compared to DEL [31].

**Figure 4:**
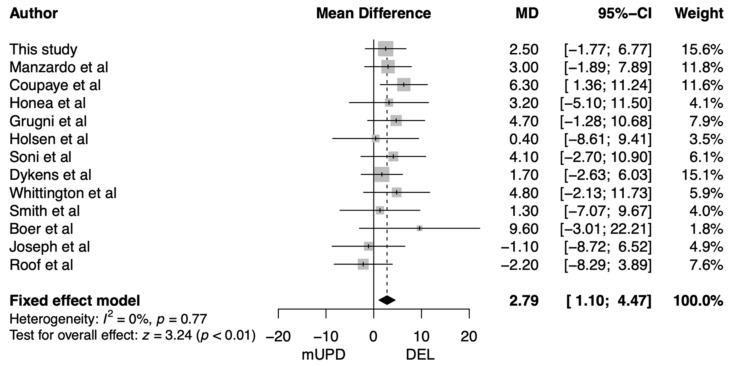
Forest plot of the difference in body mass index between adults with a paternal deletion and maternal uniparental disomy. Abbreviations: CI, confidence interval; DEL, paternal deletion; MD, mean difference; mUPD, maternal uniparental disomy.

Most studies found non-significantly higher BMI in adults with a DEL compared to mUPD. Therefore, we conducted a meta-analysis on BMI including our own data. We found that BMI was 2.79 kg/m^2^ higher in adults with a DEL compared to adults with an mUPD (95% CI: 1.10–4.47 kg/m^2^, *p* = 0.001; Figure 4).

Regarding behavioral challenges, there was no consensus on the differences between the genetic subtypes. However, it seems that adults with an mUPD might have more challenging behavior in general, but less compulsive behavior than adults with a DEL [8,17,19,34,36,38]. Although one study found more severe compulsive behavior in DEL-2 [8] and another study found less adaptive behavior in DEL-1 [17], other studies reported no differences in overall behavior between DEL-1 and DEL-2 [19,36]. However, some small differences may exist [17]. For cognition, most studies reported lower VIQ [11,15,16] and higher PIQ [11,42,48] in adults with a DEL than in adults with an mUPD. While total IQ was similar in most individual studies [15,16,42,45,46,48], a meta-analysis by Yang et al. [11] showed lower total IQ for DEL compared to mUPD. No differences in VIQ, PIQ and total IQ between DEL-1 and DEL-2 were reported [16,47]. Psychosis and bipolar disorder were more common in adults with an mUPD than DEL [11,39,44,51]. Additionally, adults with a DEL-1 might have more psychiatric diagnoses than adults with a DEL-2 [33].

## 4. Discussion

We studied genetic subtype differences in relation to health problems among 93 adults with PWS due to an mUPD or DEL. Supported by our literature overview, we conclude that differences between the genetic subtypes seem mostly present in the psychological domain. Psychotic episodes, especially, were more frequent in adults with an mUPD than in in adults with DEL. Apart from an increased frequency of scoliosis in adults with a DEL, there were no significant differences in physical health outcomes between the genetic subtypes.

In our cohort, 68% of the adults with a genetically confirmed diagnoses of PWS had a DEL, 29% an mUPD and 3% an ICD, which is similar to the previously reported distribution of genetic subtypes in PWS [6]. The size of the deletion was determined in 45 adults (69%), of whom 27% had a DEL-1, 60% a DEL-2 and 13% had an atypical microdeletion. The proportion of adults with a DEL-1 was slightly lower than in most previously reported studies [8,16,17,33,38,47]. A possible explanation for this discrepancy is that when analyzing the size of the deletion, sometimes, only the proximal BP is determined instead of both the proximal and distal BPs [7]. This can lead to inaccurate classification of deletion types and, therefore, a higher proportion of DEL-1 in previous literature. In our study, we found that 13% of the adults had an atypical microdeletion, which is higher than previously reported [6] and supports the hypothesis that some deletions classified as DEL-1 might actually be atypical microdeletions.

### 4.1. Physical Health Problems

We found that scoliosis was more prevalent in adults with a DEL compared to mUPD, even after correcting for previous GH treatment, which, in the past, has been suggested to increase the chance of scoliosis [54]. This finding was not reported in previous literature. Part of our population lived in specialized PWS group homes (20%), of which 75% had a DEL and 25% an mUPD. As caregivers in these homes have extensive PWS experience, this might have had an influence on the prevalence of scoliosis among adults with a DEL due to increased awareness and detection. However, a difference in the presence of scoliosis due to the different genetic mechanisms underlying PWS may also exist.

BMI was slightly higher in adults with a DEL compared to mUPD. Coupaye et al. [21] suggested that this difference might be due to increased adipocyte expansion in individuals with a DEL. Another explanation might be a difference in lifestyle between the two groups. More research is needed to unravel the difference in BMI between the genetic subtypes.

Smith et al. [49] reported that mortality was higher in individuals with an mUPD than in individuals with DELs. In our cohort, four patients had died, of which two had an mUPD (7%) and two had a DEL (3%; *p* = 0.38). The cause of death was unknown in one patient and of cardiovascular origin in the other patients.

### 4.2. Psychopathology, Cognition and Behavior

Similar to previous literature, we found that psychotic episodes were more prevalent in adults with an mUPD [11,51]. In our cohort, psychotic episodes were present in 44% of the adults with an mUPD. This is in agreement with previous studies, where psychotic features were present in approximately 30–60% of the adults with an mUPD [33,39,44]. A likely explanation for the increased prevalence of psychosis in adults with mUPD is overexpression of the (in the brain) paternally imprinted gene *UBE3A*. The expression of *UBE3A* is higher in persons with an mUPD than in DEL and higher than in persons without PWS [55]. *UBE3A* plays a role in the development and function of the nervous system and may indirectly alter the GABA and glutamatergic system [56,57]. Overexpression of *UBE3A* can disrupt these systems, which disturbs the excitatory–inhibitory balance in the brain, possibly leading to psychosis [57]. The hypothesis that overexpression of *UBE3A* can result in psychosis is supported by studies about patients with a 15q11-q13 duplication [58,59]. Isles et al. found that maternal duplications of this region are associated with psychosis [58]. Additionally, Noor et al. reported symptoms of psychosis in patients with maternal microduplications of *UBE3A* only [59]. Notably, both studies also reported symptoms of autism, which suggests that overexpression of *UBE3A* might also contribute to the increased prevalence of autistic characteristics in patients with an mUPD [60,61]. Other genes and snoRNAs that might play a role in the onset of psychosis are *GABRG3*, *NDN*, *CYFIP1* and *SNORD115* [57]. The most likely hypothesis is that those genes and snoRNAs are involved in a two-hit model, as previously suggested [44]. This model is based on the idea that several genetic and neural alterations are needed for the onset of psychosis [57]. Loss of expression of *GABRG3*, *NDN*, *CYFIP1* and the snoRNA cluster *SNORD115* may act as a first hit [57]. Overexpression of *UBE3A* may be the second hit, ultimately leading to psychosis in adults with an mUPD.

Several studies reported better verbal capacities and lower PIQ in adults with an mUPD compared to DEL [11,15,16,17,42,48]. We hypothesize that overexpression of *UBE3A* may contribute to the decreased performance capacities in adults with an mUPD. This is supported by a study of Baker et al. who showed that higher *UBE3A* mRNA levels were associated with lower PIQ scores [62]. However, the maternally imprinted genes may also play a role. When it comes to VIQ, not only the imprinted genes but also the non-imprinted genes in the chromosome 15q11.2-q13 region might be involved. Individuals with a DEL are hemizygous for these genes, whereas individuals with an mUPD are suspected to have normal expression. This might influence the verbal capacities in adults with a DEL. Indeed, studies on 15q11.2 BP1-BP2 microdeletions, in which the non-imprinted genes *NIPA1*, *NIPA2*, *CYFIP1* and *TUBGCP5* are deleted, showed that verbal IQ was impaired in these patients [63]. This also suggests that there might be a difference in VIQ between individuals with a DEL-1 and DEL-2, which was confirmed by Milner et al., who found higher VIQ scores in children with a DEL-2 [20]. However, other studies, among adults, did not find a difference in VIQ between the DEL subtypes [16,17].

As a post-hoc analysis, we studied the occurrence of mutism or other speech/language abnormalities in relation to the genetic subtype in our cohort. In total, five adults (four mUPD, one atypical microdeletions) had impaired speech, of which three had complete mutism. What is remarkable is that all adults with complete mutism had an mUPD. The patient with an atypical microdeletion encompassing the genes between *TUBGCP5* and *SNRPN* had a remarkable speech, speaking fluently but placing the words in the sentences in a random order. Language disorders in individuals with PWS were previously described by Dimitropoulos et al. [64]. They showed that core language ability was impaired in PWS and that both expressive and receptive language abilities were lower than VIQ. In addition, they reported that individuals with an mUPD had expressive language abilities exceeding receptive language abilities, whereas expressive and receptive language abilities did not differ within individuals with a DEL. More research is needed to elucidate the effect of the genetic subtypes on language abilities.

The studies on behavior were inconclusive. Most studies agree that compulsive behavior is more prevalent in adults with a DEL than in mUPD [8,17], but the difference between DEL-1 and DEL-2 is less clear. One study found more severe compulsive behavior in adults with a DEL-2 compared to DEL-1 [8], whereas other studies suggest that compulsive behavior might be more prevalent in DEL-1 [16,17]. Although Butler et al. [17] showed that skin picking is not associated with compulsive behavior, several parents and caregivers of PWS adults who attended our clinic do report a relation between the two (personal communication), with an increase in both skin picking and other compulsive behavior during stressful episodes. Some studies showed that skin picking, like compulsive behavior, also seems more prevalent in adults with a DEL compared to mUPD [17,19]. In our cohort, we found no significant difference in skin picking between the genetic subtypes, but small differences may exist. We hypothesize that the genes in the non-imprinted regions may contribute to the compulsive behavior in adults with a DEL. Since compulsive behavior may be more severe in adults with a DEL-1, the genes in the proximal non-imprinted region (*NIPA1*, *NIPA2*, *CYFIP1*, *TUBGCP5*) might play a significant role. In addition, Bittel et al. [65] showed that *NIPA2* and *CYFIP1* mRNA levels especially were associated with several sub-items of compulsive behavior. Moreover, variants in *TUBGCP5* are known to be related to obsessive-compulsive disorders [66]. Contrary to compulsive behavior and skin picking, other challenging behavioral phenotypes seem more prevalent in adults with an mUPD [34,36,38]. When looking at the DEL subtypes, one study reported less adaptive behavior in DEL-1 than in DEL-2 and more maladaptive behavior in DEL-2 than in mUPD [17], whereas another study reported no differences in overall behavioral profile between DEL-1 and DEL-2 [36]. In our cohort, we also found that temper outbursts tended to be more prevalent among adults with an mUPD (43%) compared to DEL (30%). However, this was not significant. We found no difference in temper outbursts between DEL-1 and DEL-2. We hypothesize that the reported challenging behavior in patients with an mUPD might be partly due to overexpression of *UBE3A*, but also other genes within the chromosome 15q11.2-q13 region are probably involved.

### 4.3. Growth Hormone Treatment

Nowadays, most people with PWS receive GH treatment during childhood, which is often continued into adulthood. Previous research showed that the positive effects of GH treatment are seen in both adults with a DEL and mUPD [67]. Coupaye et al. [21] found that the effects of previous GH treatment on reduction in body fat and adipocyte volume might be greater in people with a DEL. As expected, we noticed in our cohort that adults who had never received GH treatment had higher rates of several health problems, such as obesity. In our stratified analysis, we also show that among adults who had ever been treated with GH, scoliosis was more prevalent for DEL and psychosis was more prevalent for mUPD. No differences were detected among adults who had never received GH treatment. While this might be due to differences in effect of GH treatment between the genetic subtypes, it is most likely caused by a lack of power due to small sample sizes within the strata. However, more research is needed to elucidate the possible genetic subtype specific effects of GH treatment on health problems in adults with PWS.

### 4.4. Strengths and Limitations

Like every study, our study has strengths and limitations. A strength of our study is the relatively large cohort of adults with a confirmed genetic diagnosis of PWS. Furthermore, we used a systematic health screening to evaluate the health of all adults with PWS attending our clinic, which improved the quality of our data. Moreover, we provide a thorough overview of the current knowledge, including a meta-analysis, and relate the previous findings to our own results. However, some limitations remain. Fifteen patients were excluded from analyses because it was impossible to distinguish between mUPD and ICD due to unavailable parental genetic material. Since this group generally resembles an older population with less involved parents, this might have influenced the results. However, the age difference between adults with an mUPD and DEL is small, which suggests that the groups are still comparable. Furthermore, since additional tests for osteoporosis were only performed on indication, we had, relatively, many missing values for osteoporosis/osteopenia. We had no data on the chronicity of the psychosis, i.e., whether it was a single episode of psychotic behavior or a recurrent form of psychosis. Furthermore, the questionnaire on physical complaints, symptoms of disease and behavioral challenges was not filled out by all participants, which reduced our statistical power. Therefore, more (small) differences may exist between the genetic subtypes, and our results should be interpreted with caution.

### 4.5. Future Research

To further explore the differences in health problems between adults with an mUPD, DEL-1 and DEL-2, more research is needed. Further research should not only focus on the differences in behavior and cognition, but also on physical aspects, such as BMI and scoliosis. Moreover, differences in language abilities between the genetic subtypes should be studied. In addition, more research on atypical microdeletions is needed to further unravel the influence of the individual genes in the chromosome 15q11.2-q13 region on specific components of the PWS phenotype.

## 5. Conclusions

Differences in health problems between adults with an mUPD and DEL seem mostly present in the psychological domain. Especially, psychotic episodes were more frequent in adults with an mUPD. Apart from scoliosis and the meta-analysis on BMI, there were no significant differences in physical health outcomes between the genetic subtypes. More research is needed to further explore the differences in health problems between adults with different genetic subtypes. In addition, more data on individuals with an atypical microdeletion are needed to unravel the influence of the genes in the chromosome 15q11.2-q13 region on the PWS phenotype.

## Figures and Tables

**Table 1:** Characteristics of adults with a genetically confirmed diagnosis of Prader–Willi syndrome.

	Deletion				mUPD (N = 28)	ICD (N = 3)	mUPD or ICD (N = 15)	All Genotypes (N = 111)
	Type 1 (N = 12)	Type 2 (N = 27)	Other ^a^ (N = 26)	All Deletions (N = 65)				
Gender, N	5 M, 7 F	12 M, 15 F	12 M, 14 F	29 M, 36 F	11 M, 17 F	2 M, 1 F	9 M, 6 F	51 M, 60 F
Age in years								
Median	27.9	22.0	29.5	28.1	24.8	22.2	49.5	28.5
IQR	21.9–37.0	19.0–30.5	25.6–39.4	20.5–34.2	19.7–33.9	21.0–23.4	36.0–54.6	21.1–38.7
BMI in kg/m^2^								
Median	32.4	32.0	29.9	31.1	27.4	27.4	29.5	29.1
IQR	26.3–38.7	25.6–38.4	26.6–35.7	26.3–38.2	24.4–33.5	27.3–28.0	27.9–44.2	26.3–37.3
Current GH treatment, N (%)	5 (42%)	13 (48%)	5 (19%)	23 (35%)	16 (57%)	2 (67%)	0 (0%)	41 (37%)
GH treatment during childhood, N (%)	5 (42%)	18 (67%)	7 (27%)	30 (46%)	15 (58%) ^b^	3 (100%)	1 (7%)	49 (45%) ^b^
Psychotropic drugs, N (%) ^c^	0 (0%)	5 (19%)	7 (27%)	12 (18%)	13 (50%) ^b^	1 (33%)	13 (87%)	51 (47%) ^b^

Abbreviations: BMI, body mass index; F, female; GH, growth hormone; ICD, imprinting center defect; IQR, interquartile range; M, male; mUPD, maternal uniparental disomy. There were no significant differences for gender, age, BMI, current GH treatment and GH treatment during childhood between deletion type 1 and deletion type 2, nor between deletion and mUPD. Use of psychotropic drugs was significantly higher among adults with an mUPD (*p* = 0.002). ^a^ In 20 patients, the size of the deletion was not determined. Six patients had an atypical (micro)deletion. ^b^ In two adults with an mUPD, it was unknown whether they had received GH treatment during childhood and whether they used psychotropic drugs. ^c^ Psychotropic drugs could be prescribed for both psychosis and challenging behavior.

**Table 2:** Differences in health problems, physical complaints, symptoms of disease and behavioral challenges between adults with a deletion and maternal uniparental disomy.

	Deletion (N= 65)	Missing	mUPD (N = 28)	Missing	*p*-Value	Adjusted *p*-Value ^a^
*Health problems*						
Scoliosis ^b^	51 (80%)	1	15 (58%)	2	**0.03**	**0.04**
Osteoporosis or osteopenia	28 (54%)	13	9 (39%)	5	0.24	0.65
Osteoporosis	7 (13%)	13	2 (9%)	5	0.56	N/A
Osteopenia ^c^	21 (47%)	13	7 (33%)	5	0.31	0.63
Psychosis	6 (9%)	0	12 (44%)	1	**<0.001**	N/A
Epilepsy	3 (5%)	0	2 (7%)	0	0.62	N/A
Hypercholesterolemia ^b^	11 (17%)	1	5 (19%)	2	0.82	N/A
Type 2 diabetes mellitus ^b^	7 (11%)	3	5 (19%)	2	0.32	N/A
Overweight (BMI 25–30) ^c^	21 (68%)	0	10 (56%)	0	0.39	N/A
Obesity (BMI ≥ 30)	34 (52%)	0	10 (36%)	0	0.14	0.24
Hypertension ^b^	8 (13%)	2	7 (27%)	2	0.10	N/A
Hypothyroidism ^b^	11 (17%)	0	5 (19%)	2	0.79	N/A
*Physical complaints, symptoms of disease and behavioral challenges*						
Abdominal pain	9 (17%)	12	1 (5%)	6	0.15	N/A
Constipation	23 (37%)	3	8 (31%)	2	0.57	0.58
Diarrhea	6 (11%)	12	2 (9%)	5	0.73	N/A
Belching/heartburn	4 (8%)	14	3 (14%)	7	0.40	N/A
Vomiting	0 (0%)	12	0 (0%)	6	N/A	N/A
Urinary incontinence	2 (4%)	11	3 (14%)	7	0.10	N/A
Fecal incontinence	2 (4%)	11	0 (0%)	7	0.37	N/A
Chest pain	4 (7%)	11	0 (0%)	9	0.22	N/A
Nycturia	12 (23%)	13	3 (18%)	11	0.64	N/A
Orthopnea	0 (0%)	14	1 (5%)	9	0.10	N/A
Fatigue	13 (26%)	15	2 (10%)	7	0.12	N/A
Disturbed sleep	1 (2%)	15	1 (5%)	7	0.52	N/A
Daytime sleepiness	18 (35%)	14	7 (30%)	5	0.68	0.70
Snoring	10 (21%)	17	5 (29%)	11	0.47	N/A
Thirst	6 (12%)	15	1 (6%)	11	0.47	N/A
Somnolence or fainting during illness	2 (4%)	10	1 (4%)	5	0.88	N/A
Cold intolerance	10 (19%)	12	6 (30%)	8	0.31	N/A
Back pain	7 (13%)	13	3 (14%)	7	0.93	N/A
Bone fractures	2 (4%)	11	0 (0%)	5	0.35	N/A
Foot complaints	9 (18%)	16	9 (39%)	5	0.06	N/A
Swollen legs	8 (17%)	19	6 (30%)	8	0.25	N/A
Visual complaints	6 (12%)	15	4 (20%)	8	0.39	N/A
Temper outbursts	15 (30%)	15	10 (43%)	5	0.26	0.25
Food-seeking behavior	13 (28%)	19	7 (37%)	9	0.50	0.48
Pica	4 (8%)	14	2 (10%)	7	0.81	N/A
Skin picking	20 (42%)	17	10 (56%)	10	0.31	0.29
Sexual problems ^d^	3 (6%)	14	1 (5%)	6	0.82	N/A

Abbreviations: BMI, body mass index; mUPD, maternal uniparental disomy. Data are presented as N (%). Significant *p*-values are in bold. ^a^ Adjusted for growth hormone treatment, either currently or during childhood. ^b^ These health problems were previously studied in partly the same study population by our research group [22]. ^c^ Adults who were diagnosed with osteoporosis or obesity were excluded from the analyses for osteopenia and overweight, respectively. ^d^ Defined as hypersexuality and/or inappropriate sexual behavior.

**Table 3:** Differences in health problems between adults with a deletion and maternal uniparental disomy according to growth hormone treatment.

	Ever Treated with GH				Never Treated with GH			
	Deletion (N = 32)	mUPD (N = 17)	Missing	*p*-Value	Deletion (N = 33)	mUPD (N = 11)	Missing	*p*-Value
Scoliosis	26 (81%)	8 (53%)	0 DEL, 2 mUPD	**<0.05**	25 (78%)	7 (64%)	1 DEL, 0 mUPD	0.34
Osteoporosis or osteopenia	9 (31%)	4 (24%)	3 DEL, 0 mUPD	0.59	19 (83%)	5 (83%)	10 DEL, 5 mUPD	0.97
Osteoporosis	0 (0%)	0 (0%)	3 DEL, 0 mUPD	N/A	7 (30%)	2 (33%)	10 DEL, 5 mUPD	0.89
Osteopenia ^a^	9 (31%)	4 (24%)	3 DEL, 0 mUPD	0.59	12 (75%)	3 (75%)	10 DEL, 5 mUPD	1
Psychosis	2 (6%)	8 (50%)	0 DEL, 1 mUPD	**<0.001**	4 (12%)	4 (36%)	0 DEL, 0 mUPD	0.07
Epilepsy	1 (3%)	1 (6%)	0 DEL, 0 mUPD	0.64	2 (6%)	1 (9%)	0 DEL, 0 mUPD	0.73
Hypercholesterolemia	3 (9%)	1 (7%)	0 DEL, 2 mUPD	0.76	8 (25%)	4 (36%)	1 DEL, 0 mUPD	0.47
Type 2 diabetes mellitus	1 (3%)	1 (7%)	1 DEL, 2 mUPD	0.59	6 (19%)	4 (36%)	2 DEL, 0 mUPD	0.26
Overweight (BMI 25–30) ^a^	15 (71%)	6 (43%)	0 DEL, 0 mUPD	0.09	6 (60%)	4 (100%)	0 DEL, 0 mUPD	0.13
Obesity (BMI ≥ 30)	11 (34%)	3 (18%)	0 DEL, 0 mUPD	0.22	23 (70%)	7 (64%)	0 DEL, 0 mUPD	0.71
Hypertension	1 (3%)	3 (20%)	1 DEL, 2 mUPD	0.06	7 (22%)	4 (36%)	1 DEL, 0 mUPD	0.34
Hypothyroidism	7 (22%)	4 (29%)	0 DEL, 2 mUPD	0.72	4 (12%)	1 (9%)	0 DEL, 0 mUPD	0.78

Abbreviations: BMI, body mass index; DEL, deletion; GH, growth hormone; mUPD, maternal uniparental disomy. Data are presented as N (%). Significant *p*-values are in bold. ^a^ Adults who were diagnosed with osteoporosis or obesity were excluded from the analyses for osteopenia and overweight, respectively.

**Table 4:** Differences in health problems, physical complaints, symptoms of disease and behavioral challenges between adults with a deletion type 1 and deletion type 2.

	Type 1 (N = 12)	Missing	Type 2 (N = 27)	Missing	*p*-Value
*Health problems*					
Scoliosis	10 (83%)	0	23 (88%)	1	0.66
Osteoporosis or osteopenia	4 (36%)	1	14 (58%)	3	0.23
Osteoporosis	2 (18%)	1	1 (4%)	3	0.17
Osteopenia ^a^	2 (22%)	1	13 (57%)	3	0.08
Psychosis	1 (8%)	0	3 (11%)	0	0.79
Epilepsy	1 (8%)	0	0 (0%)	0	0.13
Hypercholesterolemia	2 (17%)	0	5 (19%)	0	0.89
Type 2 diabetes mellitus	1 (8%)	0	3 (12%)	1	0.76
Overweight (BMI: 25–30) ^a^	3 (60%)	0	8 (62%)	0	0.71
Obesity (BMI: ≥ 30)	7 (58%)	0	14 (52%)	0	0.95
Hypertension	1 (8%)	0	2 (7%)	0	0.92
Hypothyroidism	2 (17%)	0	5 (19%)	0	0.89
*Physical complaints, symptoms of disease and behavioral challenges*					
Abdominal pain	1 (10%)	2	3 (14%)	6	0.74
Constipation	3 (25%)	0	10 (40%)	2	0.37
Diarrhea	0 (0%)	2	1 (5%)	5	0.49
Belching/heartburn	1 (10%)	2	2 (10%)	7	1
Vomiting	0 (0%)	2	0 (0%)	5	N/A
Urinary incontinence	0 (0%)	2	0 (0%)	5	N/A
Fecal incontinence	0 (0%)	2	1 (5%)	5	0.49
Chest pain	1 (9%)	1	3 (14%)	5	0.71
Nycturia	2 (20%)	2	4 (20%)	5	0.90
Orthopnea	0 (0%)	2	0 (0%)	6	N/A
Fatigue	4 (40%)	2	4 (19%)	6	0.21
Disturbed sleep	0 (0%)	2	1 (5%)	1	0.46
Daytime sleepiness	3 (30%)	2	8 (38%)	6	0.66
Snoring	1 (10%)	2	4 (20%)	7	0.49
Thirst	1 (10%)	2	3 (15%)	7	0.70
Somnolence or fainting during illness	0 (0%)	1	1 (5%)	5	0.47
Cold intolerance	3 (30%)	2	3 (14%)	5	0.27
Back pain	1 (10%)	2	3 (14%)	6	0.74
Bone fractures	1 (10%)	2	1 (4%)	4	0.53
Foot complaints	2 (20%)	2	1 (5%)	8	0.22
Swollen legs	3 (33%)	3	2 (10%)	7	0.12
Visual complaints	1 (11%)	1	1 (5%)	7	0.55
Temper outbursts	3 (30%)	2	7 (33%)	6	0.85
Food-seeking behavior	3 (33%)	3	4 (22%)	9	0.53
Pica	1 (10%)	2	0 (0%)	6	0.14
Skin picking	2 (20%)	2	7 (39%)	9	0.31
Sexual problems ^b^	1 (10%)	2	0 (0%)	5	0.13

Abbreviations: BMI, body mass index. Data are presented as N (%). ^a^ Adults who were diagnosed with osteoporosis or obesity were excluded from the analyses for osteopenia and overweight, respectively. ^b^ Defined as hypersexuality and/or inappropriate sexual behavior.

**Table 5:** Differences in health problems between adults with a deletion type 1 and deletion type 2 according to growth hormone treatment.

	Ever Treated with GH			Never Treated with GH		
	DEL-1 (N = 5)	DEL-2 (N = 18)	Missing	DEL-1 (N = 7)	DEL-2 (N = 9)	Missing
Scoliosis	5 (100%)	16 (89%)	0 DEL-1, 0 DEL-2	5 (71%)	7 (88%)	0 DEL-1, 1 DEL-2
Osteoporosis or osteopenia	1 (20%)	7 (41%)	0 DEL-1, 1 DEL-2	3 (50%)	7 (100%)	1 DEL-1, 2 DEL-2
Osteoporosis	0 (0%)	0 (0%)	0 DEL-1, 1 DEL-2	2 (33%)	1 (14%)	1 DEL-1, 2 DEL-2
Osteopenia ^a^	1 (20%)	7 (41%)	0 DEL-1, 1 DEL-2	1 (25%)	6 (100%)	1 DEL-1, 2 DEL-2
Psychosis	0 (0%)	1 (6%)	0 DEL-1, 0 DEL-2	1 (14%)	2 (22%)	0 DEL-1, 0 DEL-2
Epilepsy	0 (0%)	0 (0%)	0 DEL-1, 0 DEL-2	1 (14%)	0 (0%)	0 DEL-1, 0 DEL-2
Hypercholesterolemia	1 (20%)	1 (6%)	0 DEL-1, 0 DEL-2	1 (14%)	4 (44%)	0 DEL-1, 0 DEL-2
Type 2 diabetes mellitus	0 (0%)	1 (6%)	0 DEL-1, 1 DEL-2	1 (14%)	2 (22%)	0 DEL-1, 0 DEL-2
Overweight (BMI 25–30) ^a^	3 (75%)	7 (64%)	0 DEL-1, 0 DEL-2	0 (0%)	1 (50%)	0 DEL-1, 0 DEL-2
Obesity (BMI ≥ 30)	1 (20%)	7 (39%)	0 DEL-1, 0 DEL-2	6 (86%)	7 (78%)	0 DEL-1, 0 DEL-2
Hypertension	0 (0%)	1 (6%)	0 DEL-1, 0 DEL-2	1 (14%)	1 (11%)	0 DEL-1, 0 DEL-2
Hypothyroidism	1 (20%)	4 (22%)	0 DEL-1, 0 DEL-2	1 (14%)	1 (11%)	0 DEL-1, 0 DEL-2

Abbreviations: BMI, body mass index; DEL-1, deletion type 1; DEL-2, deletion type 2; GH, growth hormone. Data are presented as N (%). ^a^ Adults who were diagnosed with osteoporosis or obesity were excluded from the analyses for osteopenia and overweight, respectively.

**Table 6:** Study characteristics of previous literature.

Author	Country	Study Design	Genotype (N)	Age in Years (Mean ± SD)	BMI in kg/m^2^ (Mean ± SD)	Gender (N)	Genetic Tests
Novell-Alsina et al. (2019) [8]	Spain	Cross- sectional	20 DEL (7 T1, 13 T2) 7 mUPD	27.30 ± 8.25	N/A	13 M, 14 F	DNA methylation, FISH, MLPA, microsatellite analysis
Debladis et al. (2019) [32]	France	Case-control (matched on sex and age)	26 DEL 13 mUPD 20 C	28.02 ± 8.03 (PWS) 24.1 ± 2.61 (C)	N/A	15 M, 24 F (PWS) 8 M, 12 F (C)	DNA methylation, FISH or QMPSF, microsatellite analysis
Manzardo et al. (2018) [33]	USA	Cross- sectional	36 DEL (14 T1, 22 T2) 29 mUPD 5 ICD	36 ± 10	28 ± 5	33 M, 37 F	MS-MLPA, high-resolution microarray, microsatellite markers
Ishii et al. (2017) [34]	Japan	Cross- sectional	35 DEL 10 mUPD	22.29 (range: 18–29)	32.58 (range: 17.29–72.23)	26 M, 19 F	FISH, “methylation test”
Coupaye et al. (2016) [21]	France	Cross- sectional	47 DEL 26 mUPD	25.5 ± 9.0	39.6 ± 10.9	35 M, 38 F	N/A
Laurier et al. (2015) [30]	France	Cross- sectional	101 DEL (16 T1, 36 T2) 42 No-DEL 3 ICD 3 Translocations 5 Unknown	28.4 ± 7.7	42.3 ± 11.1	68 M, 86 F	“methylation test”, QMPSF
Key et al. (2013) [35]	USA	Cross- sectional	13 DEL 11 mUPD	22.04 ± 5.60	N/A	12 M, 12 F	N/A
Jauregi et al. (2013) [36]	France	Cross- sectional	73 DEL (10 T1, 26 T2) 23 No-DEL 2 ICD 2 Translocations	28.2 ± 7.7	42 ± 10.6	44 M, 56 F	“Methylation test”, FISH, QMPSF
Yang et al. (2013) [11]	China	Meta- analysis	423 DEL 318 mUPD 3 Unknown	25.6 ± 10.8 ^a^	N/A	274 M, 330 F ^b^	DNA methylation, FISH, microsatellite analysis, MLPA
Honea et al. (2012) [37]	USA	Case-control (matched on age)	15 DEL (5 T1, 10 T2) 8 mUPD 25 C	22.5 ± 11.7 (PWS) 22.3 ± 10.9 (C)	31.9 ± 9.2 (PWS) 20.7 ± 2.8 (C)	8 M, 15 F (PWS) 11 M, 14 F (C)	DNA methylation, FISH, microsatellite analysis, quantitative PCR
Sinnema et al. (2011a) [38]	NL	Cross- sectional	54 DEL (23 T1, 22 T2) 42 mUPD 2 ICD	36.4 ± 12.4	32.5 ± 7.9	48 M, 50 F	DNA methylation, MLPA, microsatellite analysis
Sinnema et al. (2011b) [39]	NL	Cross- sectional	55 DEL 47 mUPD/ICD	36.2 ± 12.4	N/A	49 M, 53 F	DNA methylation, MLPA, microsatellite analysis
Sinnema et al. (2011c) [31]	NL	Cross- sectional	55 DEL 44 mUPD 3 ICD	36.2 (range: 18–66)	32.2 ± 7.9	49 M, 53 F	DNA methylation, MLPA, microsatellite analysis
Grugni et al. (2011) [40]	Italy	Cross- sectional	21 DEL (6 T1, 15 T2) 16 mUPD	26.7 ± 6.1	45.2 ± 9.7	14 M, 23 F	FISH, microsatellite analysis
Maas et al. (2010) [41]	NL	Cross- sectional	45 DEL 33 mUPD 1 ICD	34.4 ± 11.8	33.2 ± 8.0	34 M, 45 F	DNA methylation, “cytogenetic analyses”, microsatellite analysis
Copet et al. (2010) [42]	France	Cross- sectional	57 DEL 27 No-DEL	24.4 (range: 16–48)	42.6 (range: 20.8–68)	33 M, 51 F	“Methylation test”, FISH
Holsen et al. (2009) [43]	USA	Case-control (matched on age)	9 DEL (9 T2) 9 mUPD 9 C	22.4 ± 11.2 (PWS) 23.6 ± 11.9 (C)	32.3 ± 9.8 (PWS) 20.7 ± 2.3 (C)	5 M, 13 F (PWS) 3 M, 6 F (C)	DNA methylation, FISH, microsatellite analysis, quantitative PCR
Soni et al. (2008) [44]	UK	Cross- sectional	34 DEL ^c^ 85 mUPD/ICD	31.2 ± 9.6 ^d^	36.1 ± 12.1 ^d^	21 M, 25 F ^d^	DNA methylation, microsatellite analysis
Dykens et al. (2008) [19]	USA	Cross- sectional	55 DEL (26 T1, 29 T2) 33 mUPD	22.41 ± 11.74	31.3 ± 9.8	43 M, 45 F	“Methylation test”, FISH, microsatellite analysis, MS-MLPA, MLPA
Zarcone et al. (2007) [16]	USA	Cross- sectional	42 DEL (16 T1, 26 T2) 31 mUPD	22.8 ± 9.0	N/A	20 M, 45 F	High-resolution chromosome analysis, FISH, microsatellite analysis, quantitative PCR
Descheemaeker et al. (2006) [45]	Belgium	Case-control (matched on sex, age and IQ)	40 DEL 18 mUPD/ICD 1 Unknown 59 C	21.2 (PWS) (range: 2–51) 22.1 (C) (range: 2–51)	N/A	31 M, 28 F (PWS) 31 M, 28 F (C)	DNA methylation, “chromosomal examination”
Stauder et al. (2005) [46]	NL UK	Case-control (matched on sex and age)	11 DEL 11 mUPD 11 C	27.2 ± 8.1 (PWS) 27.3 ± 8.25 (C)	N/A	11 M,11 F (PWS) 6 M, 5 F (C)	N/A
Hartley et al. (2005) [47]	USA	Cross- sectional	40 DEL (14 T1, 20 T2) 23 mUPD 2 ICD	23.81 ± 8.87	34.64 ± 9.05	29 M, 36 F	High-resolution chromosome analysis, FISH, microsatellite analysis, quantitative PCR
Butler et al. (2004) [17]	USA	Cross- sectional	26 DEL (12 T1, 14 T2) 21 mUPD	23.0 ± 8.5	N/A	21 M, 26 F	DNA methylation, FISH, microsatellite analysis, quantitative PCR
Whittington et al. (2004) [48]	UK	Cross- sectional	46 DEL 25 mUPD	20.5 ± 11.8	31.6 ± 11.6	N/A	N/A
Smith et al. (2003) [49]	Australia	Prospective cohort	22 DEL 8 mUPD 1 ICD 5 Methylation +	30.8 ± 9.4	42.1 ± 10.5	18 M, 18 F	N/A
Webb et al. (2002) [50]	UK	Cross- sectional	30 DEL 13 mUPD	>18	N/A	19 M, 21 F ^e^	“Chromosome studies”, DNA methylation, “cytogenetics”, microsatellite analysis
Boer et al. (2002) [51]	UK	Cross- sectional	13 DEL 8 mUPD 1 ICD 3 Unknown	38.5 ± 5.7 ^f^	38.0 ± 3.3 ^f^	8 M, 7 F ^f^	DNA methylation, “molecular and cytogenetic techniques”
Joseph et al. (2001) [52]	USA	Cross- sectional	7 DEL 10 mUPD 9 C	24.8 ± 6.9 (PWS) 30.6 ± 13.4 (C)	31.5 ± 8.2 (PWS) 46.6 ± 9.0 (C)	8 M, 9 F (PWS) 3 M, 6 F (C)	N/A
Roof et al. (2000) [15]	USA	Cross- sectional	24 DEL 14 mUPD	22.2 ± 9.1	35.3 ± 8.8	16 M, 22 F	High-resolution chromosome analysis, FISH, microsatellite analysis

Abbreviations: BMI, body mass index; C, control group; DNA, deoxyribonucleic acid; F, female; FISH, fluorescence in situ hybridization; ICD, imprinting center defect; IQ, intelligence quotient; M, male; MLPA, multiplex ligation-dependent probe amplification; MS-MLPA, methylation specific MLPA; mUPD, maternal uniparental disomy; N/A, not available; NL, Netherlands; PCR, polymerase chain reaction; PWS, Prader–Willi syndrome; QMPSF, quantitative multiplex polymerase chain reaction of short fluorescent fragments; T1, paternal deletion type 1; T2, paternal deletion type 2; UK, United Kingdom; USA, United States of America. In one of our previous studies about PWS [22], we also compared health problems between mUPD and DEL. However, since all patients are also included in the current paper, we excluded the article from the overview. ^a^ One article that was included in the meta-analysis had no data on age. ^b^ Gender was unknown for 140 individuals. ^c^ No distinction was made between paternal deletion and balanced translocation. ^d^ Age, BMI and gender were only known for individuals with psychopathology. ^e^ Gender was unknown for three individuals. ^f^ Age, BMI and gender were only known for individuals with a suspicion of psychopathology.

**Table 7:** Differences between maternal uniparental disomy and paternal deletion according to previous literature.

Author	Outcome Parameter	Results	Remarks
Behavior, cognition, psychiatric diagnoses and brain			
Novell-Alsina et al. (2019) [8]	Compulsions (CBC, RBQ, Y-BOCS) ^a–c^	The presence of compulsive behaviors and daily repetitive behaviors was lower in individuals with mUPD than in individuals with DEL-1 or DEL-2 (*p* ≤ 0.04 for both). The percentage of individuals with severe compulsions was larger in the DEL-2 group than in the DEL-1 and mUPD group (*p* = 0.04).	Although questionnaires were administered according to the PAS-ADD-10 interview guidelines, only the CBC was especially designed for individuals with an intellectual disability.
Debladis et al. (2019) [32]	Face and emotion recognition skills: face/emotion discrimination (response time, accuracy), facial exploration (eye-tracking)	There was no difference in response time and accuracy between individuals with mUPD and DEL for face and emotion recognition (*p* ≥ 0.53 for all), although both were slower than controls (*p* < 0.001). Facial exploration was atypical for individuals with an mUPD, who had a preference for the mouth region compared to controls and DEL individuals, who mostly looked at the eye region (mUPD vs. control: *p* < 0.001).	Twelve individuals (4 mUPD, 8 DEL) were excluded from the eye-tracking analyses due to inaccurate recordings.
Manzardo et al. (2018) [33]	Psychiatric diagnoses ^d^ (DSM-IV-TR criteria)	There were no differences in the frequency of primary psychiatric diagnoses between individuals with a DEL and mUPD, nor among the DEL and mUPD subtypes. When controlled for age, the average number of psychiatric diagnoses was significantly higher for individuals with a DEL-1 (4.3 ± 1.2) compared to DEL-2 (3.6 ± 1.0; *p* = 0.03).	Only individuals who live at PWHO are included in the study. Since, for most individuals, residing at PWHO is contingent upon demonstration that the individual needs could not be met in a less restrictive environment, this population may be psychiatrically more ill.
Ishii et al. (2017) [34]	Aberrant (ABC-J), autistic (PARS), and food-related behavior (FRPQ) ^e^	The median aberrant behavior score and autistic behavior score were higher in individuals with an mUPD (aberrant behavior: 77 (IQR 40.5–91.25); autistic behavior: 21 (IQR 18.5–27.5)) than in individuals with a DEL (aberrant behavior: 27 (IQR 17–64); autistic behavior: 13 (IQR 9–18)). There was no apparent difference in the median food-related behavior score between individuals with an mUPD (35.5 (IQR 19–52)) and DEL (44 (IQR 35–51)).	Only the ABC-J and FRPQ were designed for individuals with an intellectual disability or showed robust psychometric properties in individuals with PWS. Statistical tests for the differences between mUPD and DEL were not conducted.
Key et al. (2013) [35]	Neural processing of social (faces) and nonsocial stimuli	There were no differences in accuracy and reaction time for detecting smiling faces among negative faces and nonsocial objects between individuals with an mUPD and DEL. For face vs. object processing, individuals with an mUPD showed no differentiation in brain responses between the stimuli, whereas individuals with a DEL did show differentiation. There were no differences between the genetic subtypes in brain responses to emotional content.	N/A
Jauregi et al. (2013) ^f^ [36]	Behavior (DBC-A) ^g^, hyperphagia (HQ) ^h^	The mean total DBC-A score was higher in individuals without a DEL (0.40 ± 0.2) than in individuals with a DEL (0.26 ± 0.2; *p* = 0.004). No differences were found between the genetic subtypes concerning hyperphagic behavior. No differences were found between individuals with a DEL-1 and DEL-2 for both questionnaires.	There was a negative correlation between BMI and total DBC-A score (*p* = 0.028). BMI was lower in individuals without a DEL than in individuals with a DEL (*p* = 0.0007)
Yang et al. (2013) [11]	IQ (FSIQ, VIQ, PIQ), psychosis, depression, bipolar disorder	FSIQ (MD (95% CI): -2.69 (−4.86, −0.52)) and VIQ (−7.50 (−9.75, −5.26)) were lower in individuals with a DEL than in individuals with an mUPD. PIQ was higher in individuals with a DEL (4.02 (1.13,6.91)). Individuals with a DEL are less susceptible for psychosis (OR (95% CI): 0.14 (0.08, 0.23)) and bipolar disorder (0.04 (0.01, 0.23)), compared to mUPD. Depression did not differ between the genetic subtypes.	Different instruments were used to assess IQ and psychiatric diseases.
Honea et al. (2012) ^i^ [37]	Brain volumes (GMV, WMV, CSF, TICV), eating behavior (TFEQ) ^j^	Mean global GMV was lower in individuals with a DEL (646 ± 29), compared to mUPD (695 ± 67; *p* = 0.025). TFEQ scores and global WMV, CSF and TICV did not differ between the groups.	N/A
Sinnema et al. (2011a) ^k^ [38]	Behavior (DBC-A) ^g^	The total DBC-A score was higher in individuals with an mUPD compared to DEL (*p* < 0.01). Individuals with a DEL-1 had higher scores than individuals with DEL-2.	No separate analyses for DEL-1 vs. DEL-2 were presented.
Sinnema et al. (2011b) ^k^ [39]	Psychiatric diagnoses (DBC-A ^g^, PAS-ADD ^l^, case vignettes)	Psychopathology and psychotic symptoms were more often present in individuals with an mUPD than in individuals with a DEL (*p* < 0.01 for all). For DEL, 56% of the individuals with psychopathology (17%) had a depressive illness with psychotic symptoms. For mUPD, 85% of the individuals with psychopathology (64%) had psychotic symptoms with or without affective components.	N/A
Maas et al. (2010) ^k^ [41]	Behavior (DBC-A ^g^), sleep disturbances (ESS, SA–SDQ) ^m^	There were no differences in the number of sleep disturbances and the DBC-A scores between individuals with an mUPD and DEL.	N/A
Copet et al. (2010) ^f^ [42]	IQ (WAIS-III ^n^)	Median (IQR) PIQ scores were higher among individuals with a DEL (54.0 (48.0–67.0)), compared to individuals without a DEL (50.5 (47.0–56.0); *p* = 0.0402). There were no differences in FSIQ and VIQ between the two groups. Only within individuals without a DEL, VIQ scores were higher than PIQ scores (*p* = 0.0022).	Five patients with a DEL and seven patients without a DEL were excluded from analyses.
Holsen et al. (2009) ^i^ [43]	Food motivation circuitry activity (fMRI), eating behavior (TFEQ ^k^)	TFEQ scores did not differ between individuals with an mUPD and DEL. The food motivation network activation was increased in individuals with a DEL, both pre- and post-meal, in the medial prefrontal cortex and amygdala, compared to mUPD. Individuals with an mUPD had increased activation post-meal in the dorsolateral prefrontal cortex and parahippocampal gyrus, compared to DEL.	N/A
Soni et al. (2008) ^p^ [44]	Psychiatric diagnoses (PAS-ADD ^l^, OPCRIT ^o^, case vignettes, family history and life events questionnaires)	History of psychiatric symptoms and psychotic symptoms was more frequent in individuals with an mUPD (64.7% and 61.8%, respectively) than in individuals with a DEL (28.2% and 16.5%, respectively; *p* < 0.001 for both). For individuals with a history of psychopathology, psychotic symptoms and bipolar disorder with psychotic symptoms were more frequent among individuals with an mUPD (95.5% and 50%, respectively) compared to DEL (58.3% and 0%, respectively; *p* ≤ 0.005 for both). Non-psychotic depressive illness was more prevalent in individuals with a DEL (42%), compared to mUPD (4.5%; *p* = 0.005), for individuals with a history of psychopathology. There were no differences in the prevalence of the diagnoses of depressive psychosis and schizophrenia spectrum disorders between the genetic subtypes.	N/A
Dykens et al. (2008) ^i^ [19]	Behavior (CBCL ^q^, Y-BOCS ^a^, VABS ^r^), hyperphagia (HQ ^h^), hospitalization	There were no differences in overall behavior and hyperphagia scores between the genetic subtypes mUPD and DEL. Skin picking was more common in individuals with a DEL (91%), compared to mUPD (70%; *p* < 0.01). Rectal picking was more prevalent among individuals with an mUPD (37% vs. 9%; *p* < 0.01). Individuals with an mUPD were more often psychiatrically hospitalized (55% vs. 19%, *p* < 0.05). There were no differences in behavior, hyperphagia or hospitalization between DEL-1 and DEL-2.	No separate analyses for children and adults.
Zarcone et al. (2007) ^i^ (16)	Compulsive behavior (Y-BOCS ^a^, CBC ^b^), academic achievement (WJB ^s^), IQ (WAIS-R, WISC-III), psychotropic medication (SSRI)	The overall number and severity of compulsions did not differ between DEL-1, DEL-2 and mUPD. There were some differences for sub items of the Y-BOCS and CBC. Individuals with an mUPD had higher VIQ, compared to DEL (*p* = 0.008). Academic achievement, total IQ, PIQ and use of SSRIs did not differ between the genetic subtypes.	Although the Y-BOCS has been used frequently in individuals with PWS, only the CBC has been validated for individuals with ID. No separate analyses for children and adults were presented.
Descheemaeker et al. (2006) [45]	PDD (PDD–MRScale) ^t^, IQ	The mean PDD–MRScale, percentage of PDD and IQ did not differ significantly between individuals with an mUPD and DEL. For individual PDD–MRScale items, contact problems with peers and unusual strong fears or panic reactions were more prevalent among individuals with an mUPD compared to DEL (*p* = 0.02 for both).	Unclear which instrument was used for assessing IQ. No separate analyses for children and adults were presented.
Stauder et al. (2005) [46]	Behavior and event-related brain activity (CPT-AX ^u^: reaction time, correct scores), IQ (RSPM)	Individuals with an mUPD had a longer reaction time (541 ± 170.5 ms) and less correct responses (75%, SD 22.4) than individuals with a DEL (reaction time: 398 ± 84.3 ms, *p* < 0.022; correct responses: 87%, SD 13.3). The P300 ERP peak (resembling late general inhibition processes) was lower in individuals with an mUPD (11.8 µV), compared to DEL (17.9 µV; *p* < 0.003). The N200 ERP component (resembling specific early inhibition processes) and IQ did not differ between mUPD and DEL.	N/A
Hartley et al. (2005) ^i^ [47]	Behavior (Reiss Screen) ^v^	Self-injurious and stealing behavior scores were higher among individuals with a DEL compared to mUPD (*p* = 0.011 and *p* = 0.033, respectively), as well as the number of individuals above the cut-offs for self-injurious (*p* = 0.044) and stealing behavior (*p* = 0.005). The physical depression score was higher in individuals with a DEL-1 than DEL-2 (*p* = 0.030), but there was no difference in the number of individuals above the cut-off for physical depression.	The study group included both adolescents and adults. Verbal IQ was significantly higher in individuals with an mUPD.
Butler et al. (2004) ^i^ [17]	Obsessive and compulsive behavior (Y-BOCS ^a^, CBC ^b^), adaptive and maladaptive behavior (SIB), IQ (WIS), VMI (VMIS), academic achievement (WJB-R ^s^)	Compared to DEL-1, individuals with an mUPD had significantly higher scores for some sub-items of academic achievement. There were no differences in maladaptive and adaptive behavior, compulsive behavior, visual processing and VIQ between mUPD and DEL-1. Compared to DEL-2, individuals with an mUPD had significantly less maladaptive behavior, less skin picking, worse visual processing, higher VIQ scores and lower object assembly scores. There were no differences in adaptive behavior (except for one sub-item) and academic achievement between mUPD and DEL-2. Individuals with a DEL-1 had significantly lower adaptive behavior, less control over compulsions, more interference of compulsions with social life, worse visual processing and lower scores for some sub-items of academic achievement than individuals with a DEL-2. There were no differences in maladaptive behavior (except for one sub-item) and VIQ between DEL-1 and DEL-2.	N/A
Whittington et al. (2004) ^p^ [48]	IQ (WIS), cognitive profile (Wechsler achievement battery of tests, WRAT)	Individuals with a DEL had higher PIQ scores than individuals with an mUPD (65.9 ± 9.5 vs. 61.05 ± 8.7, respectively; *p* = 0.04). For cognitive profile, individuals with an mUPD had a strength in vocabulary (*p* = 0.03) and weakness in coding (speed) (*p* = 0.03). There were no differences in FSIQ, VIQ, attainments in reading, spelling and arithmetic between the two groups.	*p*-values were not shown and were, therefore, self-calculated with an unpaired *t*-test (normal distribution was assumed). Only individuals with an IQ > 40 were included. No separate analyses for children and adults were presented.
Webb et al. (2002) ^p^ [50]	Behavior, IQ, temperature regulation, pain threshold, eye problems, sleep apnea	For behavioral characteristics, only jigsaw skills were worse in individuals with an mUPD than DEL (*p* < 0.05). The number of individuals with an IQ < 70 did not differ between the two groups. For somatic characteristics, more adults with an mUPD had poor temperature regulation, while there was no difference in the number of adults with eye problems, high pain threshold and sleep apnea.	Participant selection was (partly) based on behavioral characteristics. Therefore, behavioral characteristics were present in many participants. For some outcome measures, children and adults were not separately analyzed.
Boer et al. (2002) ^p^ [51]	Psychiatric diagnoses	Seven individuals had a major bipolar affective disorder or psychotic disorder, of which one had a DEL, one an ICD, and six an mUPD (RR mUPD vs. DEL: 8.125 (95% CI: 1.15–57.6)).	N/A
Joseph et al. (2001) ^i^ [52]	Visual recognition memory (recognition of repeated pictures)	Individuals with an mUPD recognized the most repeated pictures correctly, compared to controls and DEL (*p* < 0.04). There was no difference between recognition of food and non-food pictures. When increasing the time between two identical pictures, individuals with an mUPD recognized more repeated pictures (only food pictures) correctly than individuals with a DEL (*p* < 0.01). Although the degree of learning of the task was similar between individuals with an mUPD and DEL, individuals with an mUPD were better at retaining the information (*p* < 0.01 for food pictures; *p* = 0.05 for non-food pictures).	N/A
Roof et al. (2000) ^i^ [15]	IQ (WAIS-R, WIC-III), academic achievement (WJB-R, WRAT-3)	VIQ was higher among individuals with an mUPD (69.9 ± 6.4), compared to DEL (60.8 ± 8.6; *p* < 0.01). The difference between VIQ and PIQ within the individuals differed between individuals with an mUPD and DEL, with a higher VIQ-PIQ difference for mUPD (*p* < 0.001). Individuals with an mUPD were more likely to have VIQ exceeding PIQ (OR = 31.6). There were no differences in FSIQ, PIQ and academic achievement between the two groups.	No separate analyses for children and adults.
Health problems, metabolic parameters, body composition, GH secretion and deaths			
Coupaye et al. (2016) ^f^ [21]	Presence of scoliosis, hypogonadism, hypothyroidism, DM; metabolic parameters (fasting glycaemia, HbA1c, fasting insulin, HOMA-IR, lipids, liver enzymes); BMI; body composition (FM, LBM); adipocyte size; REE; hyperphagia ^w^; fasting total ghrelin	Hypothyroidism was more frequent among individuals with a DEL compared to mUPD (37% vs. 11%; *p* = 0.01). Mean HbA1c (*p* = 0.02), BMI (*p* = 0.02), FM (*p* = 0.04), and LBM (*p* = 0.01) were higher in individuals with a DEL than in individuals with an mUPD. There were no differences in adipocyte size, REE, hyperphagia score, fasting total ghrelin, and the frequency of scoliosis, hypogonadism and DM between the genetic subtypes.	Hypogonadism was present in nearly all subjects (DEL: 98%; mUPD: 92%).
Laurier et al. (2015) ^f^ [30]	Presence of DM, hypothyroidism, hypertension, hyperlipidemia, sleep apnea/hypoventilation, respiratory problems, scoliosis, skin picking, edema, urinary incontinence, constipation and epilepsy; BMI; medication use.	Individuals with a DEL had a higher BMI (+5.1 kg/m^2^; *p* ≤ 0.01) and were more often diagnosed with sleep apnea or hypoventilation (*p* ≤ 0.05) compared to individuals without a DEL. Individuals without a DEL more often used insulin and psychotropic medication than individuals with a DEL (*p* ≤ 0.05 for both). Other health problems did not differ between the two groups.	N/A
Sinnema et al. (2011c) ^k^ [31]	Presence of cardiovascular, respiratory, gastro-intestinal, genitourinary, endocrine, neurologic, orthopedic, dermatologic, ophthalmologic and otolaryngologic disease	Pneumonia, excessive daytime sleepiness, disturbed sleep, anemia and urinary incontinence were significantly more frequent among individuals with an mUPD compared to DEL. The predicted one-year mortality was higher for individuals with an mUPD than for individuals with a DEL (*p* = 0.03). Osteoporosis, knee problems, edema and ear problems were significantly more frequent among individuals with a DEL compared to mUPD. There were no differences in cardiovascular, neurologic and ophthalmologic disease between the genetic subtypes.	N/A
Grugni et al. (2011) [40]	GH secretion (GHRH arginine test)	Mean (SE) peak GH response and integrated GH secretion were lower in individuals with an mUPD (peak: 4.6 (1.6); integrated: 228.3 (71.6)) than in individuals with a DEL (peak: 9.1 (1.8); integrated: 514.9 (127.6); *p* < 0.005 for all). There were no differences between DEL-1 and DEL-2. IGF-I levels did not differ between the genetic subtypes.	N/A
Smith et al. (2003) [49]	Deaths	Nine individuals with a known genotype died, of which five with a DEL (56%) and 4 with an mUPD (44%). Compared to the living individuals with a known genotype (17 DEL (77%), 4 mUPD (18%)), relatively more individuals with mUPD had died (50% vs. 23%; *p* < 0.05). Cause of death did not differ between the two groups.	N/A

Abbreviations: ABC-J, Aberrant Behavior Checklist Japanese version; BMC, bone mineral content; BMD, bone mineral density; BMI, body mass index; CBC, Compulsive Behavior Checklist; CBCL, Child Behavior Checklist; CSF, cerebrospinal fluid volume; DBC-A, Developmental Behavior Checklist for Adults; DM, diabetes mellitus; ERP, event related potential; ESS, Epworth Sleepiness Scale; FM, fat mass; fMRI, functional magnetic resonance imaging; FRPQ, Food Related Problem Questionnaire; FSIQ, full scale intelligence quotient; GH, growth hormone; GHRH, growth hormone releasing hormone; GMV, gray matter volume; HbA1c, glycated hemoglobin; HOMA-IR, Homeostatic Model Assessment for Insulin Resistance; HQ, Hyperphagia Questionnaire; ID, intellectual disability; IQ, intelligence quotient; LBM, lean body mass; MVS, matching (Crichton or Mill Hill) vocabulary scale; OPCRIT, Operational Criteria Checklist for psychotic and affective illness; PARS, Pervasive Developmental Disorders Autism Society Japan Rating Scale; PAS-ADD, Psychiatric Assessment Schedule for Adults with Developmental Disabilities; PDD, pervasive developmental disorder; PDD–MRScale, pervasive developmental disorder mental retardation scale; PWS, Prader–Willi syndrome; RBQ, Repetitive Behavior Questionnaire; RCM, Raven’s Coloured Matrices; REE, respiratory energy expenditure; RR, risk ratio; RSPM, Raven’s Standard Progressive Matrices; SA–SDQ, Sleep Apnea subscale of the Sleep Disorders Questionnaire; SIB, Scales of Independent Behavior; SSRI, selective serotonin reuptake inhibitors; TFEQ, Three-Factor Eating Questionnaire; TICV, total-intracranial volume; VABS, Vineland Adaptive Behavior Scales-II; VMI, visual motor integration; VMIS, Visual Motor Integrations Scale; WAIS-III, Wechsler Adult Intelligence Scale III; WAIS-R, Wechsler Adult Intelligence Scale-Revised; WIS, Wechsler Intelligence Scale; WISC-III, Wechsler Intelligence Scale for Children-III; WJB, Woodcock-Johnson Psychoeducational Battery; WJB-R, Woodcock-Johnson Psychoeducational Battery—Revised; WMV, white matter volume; Y-BOCS, Yale–Brown Obsessive Compulsive Scale. ^a^ The Y-BOCS is a semi-structured interview and was used to assess the severity of the compulsive symptoms, based on five items: time spent doing compulsions, degree of distress due to compulsions, degree of interference in social activities, and ability to resist and control compulsions. ^b^ The CBC is designed for individuals with an intellectual disability to assess the presence of five groups of compulsive behavior: completeness, cleaning, ordering, checking and touching and deviant grooming. ^c^ The RBQ was used to assess the occurrence of repetitive behaviors, grouped in five categories: stereotype behavior, compulsive behavior, restricted preferences, insistence on sameness and repetitive speech. Answers on all questionnaires were provided by caregivers. ^d^ The primary psychiatric diagnoses were “any psychotic features”, “bipolar disorder (nonpsychotic)”, “anxiety disorder”, “major depressive disorder”, intermittent explosive disorder” and “excoriation (skin picking) disorder”. ^e^ The ABC-J was used to assess the extent of problem behaviors on five subscales: irritability and agitation, lethargy and social withdrawal, stereotypic behavior, hyperactivity and noncompliance, and inappropriate speech. The FRPQ is an informant-based questionnaire to assess food-related behavior on three subscales: preoccupation with food, impairment of satiety and other food-related negative behaviors. The PARS was used to evaluate current autistic states and consists of five subscales: interpersonal skills, communication, obsession, problematic behaviors and hypersensitivity. ^f^ Partly the same study population was probably studied by Coupaye et al. (2016), Laurier et al. (2015), Jauregi et al. (2013) and Copet et al. (2010). ^g^ The DBC-A is an assessment instrument to assess behavioral and emotional disturbance on six subscales: disruptive, self-absorbed, communication disturbance, anxiety/antisocial, social relating, depressive. ^h^ The HQ was used to assess (the severity of) food-related preoccupations and problems on three subscores: behavior, drive, severity. ^i^ Partly the same study population was probably studied by Joseph et al. (2001), Roof et al. (2000), Butler et al. (2004), Hartley et al. (2005), Zarcone et al. (2007), Dykens et al. (2008), Holsen et al. (2009) and Honea et al. (2012). ^j^ The TFEQ assess the degree of dietary restriction, eating disinhibition and hunger level. ^k^ Partly the same study population was studied by Sinnema et al. (2011a), Sinnema et al. (2011b), Sinnema et al. (2011c), Maas et al. (2010) and as the current manuscript. ^l^ The PAS-ADD is a semi-structured interview schedule for assessing psychopathology in individuals with an ID. ^m^ The ESS measures the general level of daytime sleepiness. The SA–SDQ measures the presence of sleep apnea. ^n^ The WAIS-III was used to measure IQ (FSIQ, VIQ, PIQ) and fourteen subtests (“digit span”, “information”, “vocabulary”, “arithmetic”, “comprehension”, “similarities”, “letter-number-sequencing”, “picture completion”, “picture arrangement”, “block design”, “object assembly”, “digit symbol coding”, “maNtrix reasoning”, “symbol search”). ^o^ The OPCRIT is a checklist for psychopathology. ^p^ Partly the same study population was studied by Soni et al. (2008), Whittington et al. (2004), Webb et al. (2002) and Boer et al. (2002). ^q^ The CBCL was used to assess behavioral disturbances on internalizing and externalizing domains. ^r^ The VABS was used to assess the performance of behaviors required for person or social self-sufficiency. ^s^ The WJB(-R) is a battery to measure general intellectual ability, specific cognitive abilities, scholastic aptitude, oral language and academic achievement. ^t^ The PDD–MRScale was used to screen for the spectrum of PDD on three aspects: social behavior, communication, stereotyped behavior. ^u^ The CPT-AX is a Go–No Go task to evaluate inhibitory control. ^v^ The Reiss Screen was used to assess maladaptive behavior on eight scales (aggressive behavior, autism, psychosis, paranoia, avoidant, behavioral depression, physical depression, dependent personality disorder) and six single items (drug/alcohol abuse, overactive, self-injury, sexual problem, suicidal tendencies, stealing). ^w^ Hyperphagia was assessed with the Dykens Hyperphagia Questionnaire, which measures the (severity of) food-related preoccupations and problems in PWS. The questionnaire has three subscores: hyperphagic behavior, hyperphagic drive, hyperphagic severity.

## Data Availability

Some or all datasets generated during and/or analyzed during the current study are not publicly available but are available from the corresponding author on reasonable request.

## Supplementary Materials

The following supporting information can be downloaded at: https://www.mdpi.com/article/10.3390/jcm11144033/s1, Table S1: Literature search. Table S2: Differences between maternal uniparental disomy and paternal deletion according to previous literature.
