# Supplementary material for: Health Problems in Adults with Prader–Willi Syndrome of Different Genetic Subtypes: Cohort Study, Meta-Analysis and Review of the Literature
Source: J Clin Med. 2022 Jul 12;11(14):4033. doi: 10.3390/jcm11144033 (PMC9323859; doi:10.3390/jcm11144033)
Supplement: Supplementary file 1 [file jcm-11-04033-s001.zip › jcm-1773985-supplementary.pdf]

Supplementary data

Title: Health problems in adults with Prader-Willi syndrome of different genetic subtypes: cohort study, meta-analysis and review of the literature

Authors: Anna G.W. Rosenberg, Charlotte M. Wellink, Juan M. Tellez Garcia, Karlijn Pellikaan, Denise H. Van Abswoude, Kirsten Davidse, Laura J.C.M. Van Zutven, Hennie T. Brüggenwirth, James L. Resnick, Aart J. Van Der Lely, Laura C.G. De Graaff

Corresponding author: Laura C.G. de Graaff, MD, PhD; Department of Internal Medicine-Endocrinology, Erasmus University Medical Center. Dr. Molewaterplein 40, 3015 GD Rotterdam, the Netherlands. E-mail: l.degraaff@erasmusmc.nl

Table of contents

Table S1. Search strategy **P. 3**

Table S2. Differences between maternal uniparental disomy and paternal deletion according to previous literature **P. 6**

Table S1. Search strategy

	Search 1
Embase	((('Prader Willi syndrome'/mj/exp OR (((prader*) NEAR/3 (willi*)) OR praderwilli* OR Prader$Willi*):ti,kw) AND ('genetics'/exp OR 'human genetics'/exp OR 'microdeletion'/de OR 'gene deletion'/de OR 'uniparental disomy'/de OR (genetic* OR gene OR genes OR microdelet* OR deletion* OR disom* OR isodisom*):ab,ti,kw)) OR (('Prader Willi syndrome'/exp OR (((prader*) NEAR/3 (willi*)) OR praderwilli* OR Prader$Willi*):ab,ti,kw) AND ('chromosome deletion 22q11'/de OR 'Albright syndrome'/de OR 'allan herndon dudley syndrome'/de OR 'Bardet Biedl syndrome'/de OR 'borjeson forssman lehmann syndrome'/de OR 'syndrome CHARGE'/de OR 'Cockayne syndrome'/de OR 'de Lange syndrome'/de OR 'Costello syndrome'/de OR 'cat cry syndrome'/de OR 'Dandy Walker syndrome'/de OR 'disorder of sex development'/exp OR 'Jacobsen syndrome'/de OR 'Kabuki makeup syndrome'/de OR 'Kallmann syndrome'/de OR 'myhre syndrome'/de OR 'neurofibromatosis type 1'/de OR 'Noonan syndrome'/de OR 'ohdo syndrome'/de OR '4h syndrome'/de OR 'Cowden syndrome'/de OR 'Rett syndrome'/de OR 'Rieger syndrome'/de OR 'ring chromosome 21'/de OR 'acrocephalosyndactyly'/de OR 'Silver Russell syndrome'/de OR 'Smith Magenis syndrome'/de OR 'Sotos syndrome'/de OR 'Alstrom syndrome'/de OR 'Opitz syndrome'/de OR 'tatton brown rahman syndrome'/de OR '47,XXX syndrome'/de OR 'karyotype 48,XXXX'/de OR 'Down syndrome'/de OR 'trisomy 21'/de OR 'trisomy 15'/de OR 'tuberous sclerosis complex'/de OR 'tuberous sclerosis'/de OR 'velocardiofacial syndrome'/de OR 'Williams Beuren syndrome'/de OR '48 xxyy syndrome'/de OR 'fragile X syndrome'/de OR 'fragile x associated tremor ataxia syndrome'/de OR 'xia gibbs syndrome'/de OR (((17p* OR 1q21* OR 1q25* OR 22q11* OR 7q33* OR 2p23.3* OR 16p11.2) NEAR/3 (deletion* OR microdelet*)) OR 22q11DS OR 15q11-13* OR ((adenylosuccinate* OR adenylosuccinase OR ap4) NEAR/3 (deficien*)) OR albright* OR borjeson* OR forssman* OR lehman* OR ((allan*) NEAR/3 (herndon*) NEAR/3 (dudley*)) OR ((bardet*) NEAR/3 (biedl*)) OR cockayne* OR de-lange* OR costello* OR cat-cry* OR cri-du-chat* OR crying-cat* OR CTNNB1* OR dandy-walker* OR Klinefelter* OR Turner* OR isodicentric* OR jacobsen* OR kabuki* OR kallmann* OR myhre* OR noonan* OR ohdo* OR cowden* OR hamartoma* OR rett* OR rieger* OR chotzen* OR saethre* OR magenis* OR smith-magenis* OR sotos* OR alstrom* OR opitz* OR tatton-brown* OR tetra-X OR triple-X OR velocardiofacial OR beuren* OR xxyy OR fragile-x OR fragile-x-associated-tremor* OR xia-gibb* OR ((down* OR williams OR charge* OR 4h*) NEAR/3 (syndrom* OR diseas* OR disorder* OR patient*)) OR ((disorder*) NEAR/3 (sex) NEAR/3 (developm*)) OR DSD OR idic* OR mUPD14* OR UPD14* OR ((uniparental) NEAR/3 (disom* OR isodisom*) NEAR/3 (14*)) OR ((neurofibromatos*) NEAR/3 (type-1 OR type1 OR type-I OR typeI OR 1 OR I)) OR recklinghausen* OR ((ring*) NEAR/3 (chromosom*) NEAR/3 (21)) OR acrocephalosyndactyl* OR ((silver*) NEAR/3 (russel*)) OR tetrasomy-X* OR trisomy-X* OR trisomy-21* OR trisomy-15* OR ((tuberous) NEAR/3 (scleros*)) OR ((fmr1*) NEAR/3 (premutation* OR pre-mutation*))):ab,ti,kw))) NOT ((animal/exp OR animal*:de OR nonhuman/de) NOT ('human'/exp)) NOT ([Conference Abstract]/lim AND [1900-2017]/py)
Medline ALL Ovid	(((*Prader-Willi Syndrome/ OR (((prader*) ADJ3 (willi*)) OR praderwilli*).ti,kf) AND (exp Genetics/ OR Gene Deletion/ OR Uniparental Disomy/ OR (genetic* OR gene OR genes OR microdelet* OR deletion* OR disom* OR isodisom*).ab,ti,kf.)) OR ((Prader-Willi Syndrome/ OR (((prader*) ADJ3 (willi*)) OR praderwilli*).ab,ti,kf.) AND (22q11 Deletion Syndrome/ OR Fibrous Dysplasia, Polyostotic/ OR Allan-Herndon-Dudley syndrome.rs. OR Bardet-Biedl Syndrome/ OR Borjeson-Forssman-

	Lehmann syndrome.rs. OR CHARGE Syndrome/ OR Cockayne Syndrome/ OR De Lange Syndrome/ OR Costello Syndrome/ OR Cri-du-Chat Syndrome/ OR Dandy-Walker Syndrome/ OR exp Disorders of Sex Development/ OR Jacobsen Distal 11q Deletion Syndrome/ OR Kabuki syndrome.rs. OR Kallmann Syndrome/ OR Growth mental deficiency syndrome of Myhre.rs. OR Ruvalcaba Churesigaew Myhre syndrome.rs. OR Neurofibromatosis 1/ OR Noonan Syndrome/ OR Blepharophimosis syndrome Ohdo type.rs. OR Hamartoma Syndrome, Multiple/ OR Rett Syndrome/ OR Rieger syndrome 2.rs. OR Axenfeld-Rieger syndrome.rs. OR Acrocephalosyndactylia/ OR Silver-Russell Syndrome/ OR Smith-Magenis Syndrome/ OR Sotos Syndrome/ OR Alstrom Syndrome/ OR Opitz GBBB Syndrome, X-Linked.rs. OR Triple X syndrome.rs. OR Tetrasomy X.rs. OR Down Syndrome/ OR Chromosome 15q, trisomy.rs. OR Chromosome 15, trisomy mosaicism.rs. OR Tuberous Sclerosis/ OR DiGeorge Syndrome/ OR Williams Syndrome/ OR Klinefelter Syndrome/ OR Fragile X Syndrome/ OR Fragile X Tremor Ataxia Syndrome.rs. OR (((17p* OR 1q21* OR 1q25* OR 22q11* OR 7q33* OR "2p23.3"* OR "16p11.2"*) ADJ3 (deletion* OR microdelet*)) OR 22q11DS OR 15q11-13* OR ((adenylosuccinate* OR adenylosuccinase OR ap4) ADJ3 (deficien*)) OR albright* OR borjeson* OR forssman* OR lehman* OR ((allan*) ADJ3 (herndon*) ADJ3 (dudley*)) OR ((bardet*) ADJ3 (biedl*)) OR cockayne* OR de-lange* OR costello* OR cat-cry* OR cri-du-chat* OR crying-cat* OR CTNNB1* OR dandy-walker* OR Klinefelter* OR Turner* OR isodicentric* OR jacobsen* OR kabuki* OR kallmann* OR myhre* OR noonan* OR ohdo* OR cowden* OR hamartoma* OR rett* OR rieger* OR chotzen* OR saethre* OR magenis* OR smith-magenis* OR sotos* OR alstrom* OR opitz* OR tatton-brown* OR tetra-X OR triple-X OR velocardiofacial OR beuren* OR xxyy OR fragile-x OR fragile-x-associated-tremor* OR xia-gibb* OR ((down* OR williams OR charge* OR 4h*) ADJ3 (syndrom* OR diseas* OR disorder* OR patient*)) OR ((disorder*) ADJ3 (sex) ADJ3 (developm*)) OR DSD OR idic* OR mUPD14* OR UPD14* OR ((uniparental) ADJ3 (disom* OR isodisom*) ADJ3 (14*)) OR ((neurofibromatos*) ADJ3 (type-1 OR type1 OR type-I OR typeI OR 1 OR I)) OR recklinghausen* OR ((ring*) ADJ3 (chromosom*) ADJ3 (21)) OR acrocephalosyndactyl* OR ((silver*) ADJ3 (russel*)) OR tetrasomy-X* OR trisomy-X* OR trisomy-21* OR trisomy-15* OR ((tuberous) ADJ3 (scleros*)) OR ((fmr1*) ADJ3 (premutation* OR pre-mutation*))).ab,ti,kf.))) NOT (exp Animals/ NOT Humans/) NOT ((news OR congres* OR abstract* OR book* OR chapter* OR dissertation abstract*).pt. AND 1900:2017.(sa_year).)
Cochrane Central Register of Controlled Trials	(((((prader*) NEAR/3 (willi*)) OR praderwilli* OR Prader$Willi*):ti) AND ((genetic* OR gene OR genes OR microdelet* OR deletion* OR disom* OR isodisom*):ab,ti)) OR (((((prader*) NEAR/3 (willi*)) OR praderwilli* OR Prader$Willi*):ab,ti) AND ((((17p* OR 1q21* OR 1q25* OR 22q11* OR 7q33* OR "2p23.3" OR "16p11.2") NEAR/3 (deletion* OR microdelet*)) OR 22q11DS OR "15q11-13" OR ((adenylosuccinate* OR adenylosuccinase OR ap4) NEAR/3 (deficien*)) OR albright* OR borjeson* OR forssman* OR lehman* OR ((allan*) NEAR/3 (herndon*) NEAR/3 (dudley*)) OR ((bardet*) NEAR/3 (biedl*)) OR cockayne* OR de-lange* OR costello* OR cat-cry* OR cri-du-chat* OR crying-cat* OR CTNNB1* OR dandy-walker* OR Klinefelter* OR Turner* OR isodicentric* OR jacobsen* OR kabuki* OR kallmann* OR myhre* OR noonan* OR ohdo* OR cowden* OR hamartoma* OR rett* OR rieger* OR chotzen* OR saethre* OR magenis* OR smith-magenis* OR sotos* OR alstrom* OR opitz* OR tatton-brown* OR tetra-X OR triple-X OR velocardiofacial OR beuren* OR xxyy OR fragile-x OR fragile-x-associated-tremor* OR xia-gibb* OR ((down* OR williams OR charge* OR 4h*) NEAR/3 (syndrom* OR diseas* OR disorder* OR patient*)) OR ((disorder*) NEAR/3 (sex) NEAR/3 (developm*)) OR DSD OR idic* OR mUPD14* OR UPD14* OR ((uniparental) NEAR/3 (disom* OR isodisom*) NEAR/3 (14*)) OR ((neurofibromatos*) NEAR/3 (type-1 OR type1 OR type-I OR typeI OR 1 OR I)) OR recklinghausen* OR ((ring*) NEAR/3 (chromosom*) NEAR/3 (21)) OR

<table>
<tr><td></td><td>acrocephalosyndactyl* OR ((silver*) NEAR/3 (russel*)) OR tetrasomy-X* OR trisomy-X* OR trisomy-21* OR trisomy-15* OR ((tuberous) NEAR/3 (scleros*)) OR ((fmr1*) NEAR/3 (premutation* OR pre-mutation*))):ab,ti))</td></tr>
<tr><td>Web of Science Core Collection</td><td>(((TI=(((prader*) NEAR/2 (willi*)) OR praderwilli* OR Prader$Willi*)) AND TS=((genetic* OR gene OR genes OR microdelet* OR deletion* OR disom* OR isodisom*))) OR TS=(((((prader*) NEAR/2 (willi*)) OR praderwilli* OR Prader$Willi*)) AND ((((17p* OR 1q21* OR 1q25* OR 22q11* OR 7q33* OR "2p23.3" OR "16p11.2") NEAR/2 (deletion* OR microdelet*)) OR 22q11DS OR "15q11-13" OR ((adenylosuccinate* OR adenylosuccinase OR ap4) NEAR/2 (deficien*)) OR albright* OR borjeson* OR forssman* OR lehman* OR ((allan*) NEAR/2 (herndon*) NEAR/2 (dudley*)) OR ((bardet*) NEAR/2 (biedl*)) OR cockayne* OR de-lange* OR costello* OR cat-cry* OR cri-du-chat* OR crying-cat* OR CTNNB1* OR dandy-walker* OR Klinefelter* OR Turner* OR isodicentric* OR jacobsen* OR kabuki* OR kallmann* OR myhre* OR noonan* OR ohdo* OR cowden* OR hamartoma* OR rett* OR rieger* OR chotzen* OR saethre* OR magenis* OR smith-magenis* OR sotos* OR alstrom* OR opitz* OR tatton-brown* OR tetra-X OR triple-X OR velocardiofacial OR beuren* OR xxyy OR fragile-x OR fragile-x-associated-tremor* OR xia-gibb* OR ((down* OR williams OR charge* OR 4h) NEAR/2 (syndrom* OR diseas* OR disorder* OR patient*)) OR ((disorder*) NEAR/2 (sex) NEAR/2 (developm*)) OR DSD OR idic* OR mUPD14* OR UPD14* OR ((uniparental) NEAR/2 (disom* OR isodisom*) NEAR/2 (14)) OR ((neurofibromatos*) NEAR/2 (type-1 OR type1 OR type-I OR typeI OR 1 OR I)) OR recklinghausen* OR ((ring*) NEAR/2 (chromosom*) NEAR/2 (21)) OR acrocephalosyndactyl* OR ((silver*) NEAR/2 (russel*)) OR tetrasomy-X* OR trisomy-X* OR trisomy-21* OR trisomy-15* OR ((tuberous) NEAR/2 (scleros*)) OR ((fmr1*) NEAR/2 (premutation* OR pre-mutation*))))))</td></tr>
<tr><td>Google Scholar</td><td>"prader willi" genetic|gene|genes|microdeletion|deletion|disomy|isodisomy|alstrom|opitz|tatton-brown|tetra-X|triple-X|velocardiofacial|beuren|xxyy|DSD|idic|mUPD14|UPD14|recklinghausen||acrocephalosyndactyl|tetrasomy-X|trisomy-X|trisomy-21|trisomy-15
"prader willi" albright|borjeson|forssman|lehman|herndon|dudley|bardet|biedl|cockayne|lange|costello|"cat cry|crying"|CTNNB1|dandy|Klinefelter|isodicentric|jacobsen|kabuki|kallmann|myhre|noonan|ohdo|cowden|hamartoma|rett |rieger|chotzen|saethre|sotos</td></tr>
</table>

Table S2. Differences between maternal uniparental disomy and paternal deletion according to previous literature

Author	Outcome parameter	Results	Remarks
		Behavior, cognition, psychiatric diagnoses and brain	
Novell-Alsina et al (2019)	Compulsions (CBC, RBQ, Y-BOCS)[a-c]	The presence of compulsive behaviors was lower in individuals with mUPD (33.3%), than in individuals with DEL-1 (85.7%) or DEL-2 (91.7%; overall *P* = .03). The daily occurrence of repetitive behaviors was lower in individuals with mUPD (28.6%), compared to individuals with DEL-1 (71.4%) or DEL-2 (84.6%; overall *P* = .04). The mUPD group showed a negative association with the presence of compulsive behaviors (OR = .06; 95% CI = .01 - .55) and with the daily occurrence of repetitive behaviors (OR = .10; 95% CI = .01 - .75) and daily compulsive behaviors (OR = .02; 95% CI = .01 - .32). The percentage of individuals with severe compulsions was larger in the DEL-2 group (76.9%) than in the DEL-1 (28.6%) and mUPD group (28.6%, overall *P* = .04). More specific, compared to DEL-1 and mUPD, a larger percentage of individuals in the DEL-2 group spent time on compulsions and had no control over their compulsions (for both: 76.9% in DEL-2 vs. 28.6% in both DEL-1 and mUPD; *P* = .04). Individuals with DEL-2 showed a positive association with severity, time, and lack of resistance (for all: OR = 8.33; 95% CI = 1.47 – 47.23), and low control over compulsions (OR = 8.25; 95% CI = 1.45 – 46.86) compared to individuals with DEL-1 and mUPD.	Although questionnaires were administered according to the PAS-ADD-10 interview guidelines, only the CBC was especially designed for individuals with an intellectual disability.
Debladis et al (2019)	Face and emotion recognition skills: face/emotion discrimination (response time, accuracy), facial exploration (eye-tracking), gaze fixation in social context (video task)	There was no difference in response time between individuals with mUPD and DEL for face (*P* = .53) and emotion recognition (*P* = .95), although both were slower than controls (*P* < .001). Similarly, individuals with PWS had a deficit in accurately recognizing faces and emotions compared to controls (*P* < .001), but this did not differ between mUPD and DEL individuals (*P* = .62 for face, *P* = .74 for emotion). Facial exploration was atypical for individuals with an mUPD, who had a preference for the mouth region, compared to controls and DEL individuals who mostly looked at the eye region (mUPD vs control: *P* < .001). The gaze fixation became more atypical for both subtypes of PWS as the social content increased.	Twelve individuals (4 mUPD, 8 DEL) were excluded from the eye-tracking analyses due to inaccurate recordings.<br><br>The video task was performed by only 24 individuals (8 mUPD, 8 DEL, 8 control).
Manzardo et al (2018)	Psychiatric diagnoses[d] (DSM-IV-TR criteria)	There were no differences in the frequency of primary psychiatric diagnoses between individuals with a DEL and mUPD, nor among the DEL and mUPD subtypes. When controlled for age, the average number of psychiatric diagnoses was significantly higher for individuals with a DEL-1 (4.3 ± 1.2) compared to DEL-2 (3.6 ± 1.0; *P* = .03)	Only individuals who live at PWHO are included in the study. Since, for most individuals, residing at PWHO is contingent upon demonstration that the individual needs could not be met in a less restrictive environment, this population may be psychiatrically more ill.

Ishii et al (2017)	Aberrant (ABC-J), autistic (PARS), and food-related behavior (FRPQ)[e]	The median aberrant behavior score was higher in individuals with an mUPD (77; IQR 40.5 – 91.25) than in individuals with a DEL (27; IQR 17 – 64). There was no apparent difference in the median food-related behavior score between individuals with an mUPD (35.5; IQR 19 – 52) and DEL (44; IQR 35 – 51). The median autistic behavior score was higher in individuals with an mUPD (21; IQR 18.5 – 27.5), compared to DEL (13; IQR 9 – 18).	Only the ABC-J and FRPQ were designed for individuals with an intellectual disability or showed robust psychometric properties in individuals with PWS.<br><br>Statistical tests for the differences between mUPD and DEL were not conducted.
Key et al (2013)	Neural processing of social (faces) and nonsocial stimuli	There were no differences in accuracy and reaction time for detecting smiling faces among negative faces and nonsocial objects between individuals with an mUPD and DEL. For face vs. object processing, individuals with an mUPD showed no differentiation in brain responses between the stimuli, whereas individuals with a DEL did show differentiation. There were no differences between the genetic subtypes in brain responses to emotional content.	N/A
Jauregi et al (2013)	Behavioral and emotional disturbances (DBC-A)[f], hyperphagia (HQ)[g]	The mean total DBC-A score was higher in individuals without a DEL (0.40 ± 0.2) than in individuals with a DEL (0.26 ± 0.2; P = .004). For the DBC-A subscales, only self-absorbed, communication disturbance, and social relation were increased in adults without a DEL, compared to DEL (P = .004, P = .001, P = .012, respectively). No differences were found between the genetic subtypes concerning hyperphagic behavior. No differences were found between individuals with a DEL-1 and DEL-2 for both questionnaires.	There was a negative correlation between BMI and total DBC-A score (P = .028), self-absorbed (P = .031), and social relating (P = .021). BMI was lower in individuals without a deletion than in individuals with a deletion (P = .0007)<br><br>Probably partly the same study population as Coupaye et al (2016), Laurier et al (2015) and Copet et al (2010).
Yang et al (2013)	IQ (FSIQ, VIQ, PIQ), psychosis, depression, bipolar disorder	FSIQ (MD [95% CI]: -2.69 [-4.86, -0.52]) and VIQ (-7.50 [-9.75, -5.26]) were lower in individuals with a DEL than in individuals with an mUPD. PIQ was higher in individuals with a DEL (4.02 [1.13,6.91]). Individuals with an mUPD are more susceptible for psychosis (OR [95% CI]: 0.14 [0.08, 0.23]) and bipolar disorder (0.04 [0.01, 0.23]), compared to individuals with a DEL. Depression did not differ between the genetic subtypes.	Different instruments were used to assess IQ and psychiatric diseases.
Honea et al (2012)	Brain volumes (GMV, WMV, CSF, TICV), eating behavior (TFEQ)[h]	Mean global GMV was lower in individuals with a DEL (646 ± 29), compared to mUPD (695 ± 67; P = .025). Global WMV, CSF and TICV did not differ between the groups. Compared to mUPD, individuals with a deletion had less GMV in the left middle frontal gyrus (P = .067), right superior temporal gyrus (P = .013), and left inferior parietal cortex (P = .003). They also had less WMV in the left inferior parietal cortex (P ≤ .024). Individuals with an mUPD had less WMV in	Probably partly the same study population as Joseph et al (2001), Roof et al (2000), Butler et al (2004), Hartley et al (2005), Zarcone et al (2007), Dykens et al (2008) and Holsen et al (2009).

		the orbitofrontal cortex (P = .021), compared to DEL. There were no differences in TFEQ scores between the genetic subtypes.	
Sinnema et al (2011a)	Behavior (DBC-A)[f]	The total DBC-A score differed between individuals with an mUPD and DEL (P < .01), with higher scores for mUPD. Individuals with a DEL-1 had higher scores than individuals with a DEL-2. For the subscales, only disruptive, self-absorbed, social relating, and depressive differed between the genetic subtypes (P < .05 for all). The scores were higher for individuals with an mUPD than for individuals with a DEL-1 or DEL-2, except for the subscale social relating, where their score was lower. On the following DBC-A items, individuals with an mUPD scored higher than individuals with a DEL: unhappy, abusive, poor attention span, facial twitches, flicks, impatient, overactive, screams a lot, soils outside the toilet, whispers, strips of clothes, tells lies, wanders aimlessly (P ≤ .05).	No separate analyses for DEL-1 vs. DEL-2 were presented.<br><br>Partly the same study population as Sinnema et al (2011b), Sinnema et al (2011c), Maas et al (2010) and as the current manuscript.
Sinnema et al (2011b)	Psychiatric diagnoses (DBC-A[f], PAS-ADD[i], case vignettes)	Psychopathology and psychotic symptoms were more often present in individuals with an mUPD than in individuals with a DEL (P < .01 for all). For DEL, 56% of the individuals with psychopathology (17%) had a depressive illness with psychotic symptoms. For mUPD, 85% of the individuals with psychopathology (64%) had psychotic symptoms with or without affective components.	Partly the same study population as Sinnema et al (2011a), Sinnema et al (2011c), Maas et al (2010) and as the current manuscript.
Maas et al (2010)	Behavior (DBC-A[f]), sleep disturbances (ESS, SA-SDQ)[j]	There were no differences in the number of sleep disturbances and the DBC-A scores between individuals with an mUPD and DEL.	Partly the same study population as Sinnema et al (2011a, 2011b, 2011c) and as the current manuscript.
Copet et al (2010)	IQ (WAIS-III[k])	Median [IQR] PIQ scores were higher among individuals with a DEL (54.0 [48.0 – 67.0]), compared to no-DEL (50.5 [47.0 – 56.0]; P = .0402). There were no differences in FSIQ and VIQ between the two groups. Only within individuals without a DEL, VIQ scores were higher than PIQ scores (P = .0022). For the subtests, individuals with a DEL had higher scores for "picture arrangement", "object assembly" and "digit symbol coding" than individuals without a DEL (P = .003, P = .044 and P = .036, respectively. Within individuals with a DEL, scores for the subtests "comprehension" (P = .017) and "picture completion" (P = .032) were relatively low and scores for "object assembly" (P = .036) were relatively high. Within individuals without a DEL, scores for "picture arrangement" (P = .007) and "matrix reasoning" (P = .046) were relatively low, whereas scores for "information" (P = .025) and "similarities" (P = .010) were relatively high. Scores for "digit span", "digit symbol coding" and "symbol search" were relatively low within both groups (P ≤ .012 for all), and scores for "vocabulary" were relatively high within both groups (P ≤ .040 for both).	Five patients with a DEL and 7 patients without a DEL were excluded from analyses.<br><br>Probably partly the same study population as Coupaye et al (2016), Laurier et al (2015) and Jauregi et al (2013).
Holsen et al (2009)	Food motivation circuitry activity (fMRI), eating behavior (TFEQ[h])	TFEQ scores did not differ between individuals with an mUPD and DEL. The food motivation network activation was increased in individuals with a DEL both pre- and post-meal in the medial prefrontal cortex and amygdala, compared to	Probably partly the same study population as Joseph et al (2001), Roof et al (2000), Butler et al (2004), Hartley et al (2005), Zarcone et al

		mUPD. Individuals with an mUPD had increased activation post-meal in the dorsolateral prefrontal cortex and parahippocampal gyrus, compared to DEL.	(2007), Dykens et al (2008) and Honea et al (2012).
Soni et al (2008)	Psychiatric diagnoses (PAS-ADD[i] OPCRIT[l], case vignettes, family history and life events questionnaires)	History of psychiatric symptoms was more frequent in individuals with an mUPD (64.7%) than in individuals with a DEL (28.2%, *P* < .001). For individuals with a history of psychopathology, psychotic symptoms were more frequent among individuals with an mUPD (95.5%) compared to DEL (58.3%, *P* = .005). Non-psychotic depressive illness was more prevalent in individuals with a DEL (42%, compared to mUPD (4.5%; *P* = .005), for individuals with a history of psychopathology. Bipolar disorder with psychotic symptoms was more prevalent among individuals with an mUPD (50% vs. 0%; *P* = .00005), for individuals with a history of psychopathology. There were no differences in the prevalence of the diagnoses depressive psychosis and schizophrenia spectrum disorders between the genetic subtypes. For the total population (with and without history of psychopathology), psychotic symptoms were also more frequent in individuals with an mUPD (61.8%) compared to DEL (16.5%; OR 8.2 [95% CI 3.3-20.1]; *P* < .001).	Partly the same study population as Whittington et al (2004), Webb et al (2002) and Boer et al (2002).
Dykens et al (2008)	Behavior (CBCL[m], Y-BOCS[a], VABS[n]), hyperphagia (HQ[g]), hospitalization	There were no differences in overall behavior and hyperphagia scores between the genetic subtypes mUPD and DEL. Skin picking was more common in individuals with a DEL (91%), compared to mUPD (70%; *P* < .01). Rectal picking was more prevalent among individuals with an mUPD (37% vs. 9%; *P* < .01). Individuals with an mUPD were more often psychiatrically hospitalized (55% vs. 19%, *P* < .05). There were no differences in behavior, hyperphagia or hospitalization between DEL-1 and DEL-2. When studying separate genetic subtypes, advancing age was associated with less hyperphagia and challenging behavior for individuals with a DEL-1 only.	No separate analyses for children and adults.<br><br>Probably partly the same study population as Joseph et al (2001), Roof et al (2000), Butler et al (2004), Hartley et al (2005), Zarcone et al (2007), Holsen et al (2009) and Honea et al (2012).
Zarcone et al (2007)	Compulsive behavior (Y-BOCS[a], CBC[b]), academic achievement (WJB[o]), IQ (WAIS-R, WISC-III), psychotropic medication (SSRI)	The overall number and severity of compulsions did not differ between DEL and mUPD. Compared to DEL-2 and mUPD, compulsions were more likely to interfere with social activities for individuals with a DEL-1 (*P* = .002), and interrupting compulsions were more likely to make them upset (*P* = .02). Individuals with an mUPD were more likely to resume the compulsive activity when interrupted (*P* = .025). For the separate Y-BOCS items, individuals with a DEL-1 had more severe compulsions regarding excessive bathing/grooming (*P* =.02) compared to DEL-2 and mUPD. Individuals with a DEL-2 had more severe repeating compulsions regarding excessively counting of numbers and objects (*P* = .04). Academic achievement, total IQ, PIQ and use of SSRIs did not differ between the genetic subtypes. Individuals with an mUPD had higher VIQ, compared to DEL (*P* = .008).	Although the Y-BOCS has been used frequently in individuals with PWS, only the CBC has been validated for individuals with ID.<br><br>No separate analyses for children and adults.<br><br>Probably partly the same study population as Joseph et al (2001), Roof et al (2000), Butler et al (2004), Hartley et al (2005), Dykens et al (2008), Holsen et al (2009) and Honea et al (2012).

Descheemaeker et al (2006)	PDD (PDD-MRScale)[p], IQ	The mean PDD-MRScale, percentage of PDD, and IQ did not differ significantly between individuals with an mUPD and DEL. For individual PDD-MRScale items, contact problems with peers and unusual strong fears or panic reactions were more prevalent among individuals with an mUPD compared to DEL (*P* = .02 for both).	Unclear which instrument was used for assessing IQ.<br><br>No separate analyses for children and adults.
Stauder et al (2005)	Behavior and event-related brain activity (CPT-AX[q]: reaction time, correct scores), IQ (RSPM)	Individuals with an mUPD had a longer reaction time than individuals with a DEL (541 ± 170.5 ms vs. 398 ± 84.3 ms; *P* < .022). Individuals with an mUPD had 75% (SD 22.4) correct responses, whereas individuals with a DEL had 87% (SD 13.3) correct responses. The P300 ERP peak (resembling late general inhibition processes) was lower in individuals with an mUPD (11.8 μV), compared to DEL (17.9 μV; *P* < .003). The N200 ERP component (resembling specific early inhibition processes) did not differ between mUPD and DEL. IQ did not differ between DEL and mUPD.	N/A
Hartley et al (2005)	Behavior (Reiss Screen)[r]	Self-injurious and stealing behavior scores were higher among individuals with a DEL compared to mUPD (*P* = .011 and *P* = .033, respectively). The number of individuals above the cut-offs for self-injurious and stealing behavior were also higher among individuals with a DEL compared to mUPD (*P* = .044 and *P* = .005, respectively). The physical depression score was higher in individuals with a DEL-1 than DEL-2 (*P* = .030). However, when looking at the number of individuals above the cut-off for physical depression, there was no difference between the groups.	The study group included both adolescents and adults.<br><br>Verbal IQ was significantly higher in individuals with an mUPD.<br><br>Probably partly the same study population as Joseph et al (2001), Roof et al (2000), Butler et al (2004), Zarcone et al (2007), Dykens et al (2008), Holsen et al (2009) and Honea et al (2012).
Butler et al (2004)	Obsessive and compulsive behavior (Y-BOCS[a], CBC[b]), psychiatric symptoms (Reiss[r]), adaptive and maladaptive behavior (SIB), IQ (WIS), VMI (VMIS), academic achievement (WJB-R[o])	Overall, individuals with a DEL-2 had more maladaptive behavior than individuals with an mUPD (*P* ≤ .048 for all sub-items). There was no difference in maladaptive behavior scores between individuals with a DEL-1 and mUPD. When comparing DEL-1 and DEL-2, only the sub-item externalized maladaptive index (from SIB) differed, with a higher score for DEL-2 compared to DEL-1 (*P* = .019). For adaptive behavior, there was no difference between individuals with an mUPD and DEL-1 or DEL-2, except for the sub-item personal living skills (from SIB). The score for personal living skills was higher in individuals with a DEL-2 than in individuals with an mUPD (*P* = .031). When comparing DEL-1 and DEL-2, adaptive behavior scores were lower in individuals with a DEL-1 (*P* ≤ .027 for all sub-items). For obsessive-compulsive behavior, individuals with an mUPD had a lower score for the sub-item skin picking and a higher score for the sub-item interruption response-halts and resumes, compared to DEL-2 (*P* ≤ .045 for both). There were no differences between mUPD and DEL-1. When comparing DEL-1 and DEL-2, individuals with a DEL-1	Probably partly the same study population as Joseph et al (2001), Roof et al (2000), Hartley et al (2005), Zarcone et al (2007), Dykens et al (2008), Holsen et al (2009) and Honea et al (2012).

		had higher scores for the sub-items control over compulsion and interference with social than individuals with a DEL-2 (*P* ≤ .021 for both).<br>For visual processing, there was no difference between individuals with a DEL-1 and mUPD. However, individuals with a DEL-2 had higher scores for the sub-items Vineland Motor Inventory percentile and Vineland Motor inventory standard score than individuals with an mUPD (*P* ≤ .024 for both). When comparing DEL-1 and DEL-2, visual processing scores were higher for DEL-2 individuals (*P* ≤ .045 for all sub-items).<br>For academic achievement, there was no difference between mUPD and DEL-2, whereas individuals with a DEL-1 had lower scores than individuals with an mUPD for the sub-items Woodcock-Johnson reading cluster, Woodcock-Johnson math cluster, and applied problems (*P* ≤ .040 for all). When comparing DEL-1 and DEL-2, individuals with a DEL-1 had lower scores for the sub-items letter-word identification, reading comprehension, woodcock-Johnson math cluster, and calculation (*P* ≤ .048 for all).<br>For IQ, there were no differences between DEL-1 and mUPD and between DEL-1 and DEL-2. Individuals with an mUPD did differ from DEL-2 with higher scores for all sub-items (*P* ≤ .030), except for the sub-item object assembly. The score for this sub-item was lower in individuals with an mUPD compared to DEL-2 (*P* < .038).	
Whittington et al (2004)	IQ (WIS), cognitive profile (Wechsler achievement battery of tests, WRAT)	Individuals with a DEL had higher PIQ scores than individuals with an mUPD (65.9 ± 9.5 vs. 61.05 ± 8.7, respectively; *P* = .0381). Individuals with an mUPD had a higher score for the subscale vocabulary and a lower score for the subscale coding, compared to DEL (*P* = .0269 and *P* = .0349, respectively). There were no differences in FSIQ, VIQ, attainments in reading, spelling and arithmetic, and in the other verbal and non-verbal subscales between the two groups.	P-values were not shown and therefore self-calculated with an unpaired t-test (normal distribution was assumed).<br><br>Partly the same study population as Soni et al (2008), Webb et al (2002) and Boer et al (2002).<br><br>No separate analyses for children and adults.
Webb et al (2002)	Behavior, IQ, temperature regulation, pain threshold, eye problems, sleep apnea	For behavioral characteristics, only jigsaw skills were worse in individuals with an mUPD than DEL (*P* < .05). The number of individuals with an IQ < 70 did not differ between the two groups. For somatic characteristics, more adults with an mUPD had poor temperature regulation, while there was no difference in the number of adults with eye problems, high pain threshold and sleep apnea.	Participant selection was (partly) based on behavioral characteristics. Therefore, behavioral characteristics were present in many participants.<br><br>For some outcome measures children and adults were not separately analyzed.

			Partly the same study population as Soni et al (2008), Whittington et al (2004) and Boer et al (2002).
Boer et al (2002)	Psychiatric diagnoses	Seven individuals had a major bipolar affective disorder or psychotic disorder, of which 1 had a DEL, 1 an ICD, and 6 an mUPD (RR mUPD vs. DEL: 8.125 [95% CI: 1.15 – 57.6]).	Partly the same study population as Soni et al (2008), Whittington et al (2004) and Webb et al (2002).
Joseph et al (2001)	Visual recognition memory (recognition of repeated pictures)	Individuals with an mUPD recognized the most repeated pictures correctly, compared to controls and DEL (*P* < .04). There was no difference between recognition of food and non-food pictures. When increasing the time between two identical pictures, individuals with an mUPD recognized more repeated pictures (only food pictures) correctly than individuals with a DEL (*P* < .01). Although the degree of learning of the task was similar between individuals with an mUPD and DEL, individuals with an mUPD were better at retaining the information (*P* < .01 for food pictures; *P* = .05 for non-food pictures).	Probably partly the same study population as Roof et al (2000), Butler et al (2004), Hartley et al (2005), Zarcone et al (2007), Dykens et al (2008), Holsen et al (2009) and Honea et al (2012).
Roof et al (2000)	IQ (WAIS-R, WIC-III), academic achievement (WJB-R, WRAT-3)	VIQ was higher among individuals with an mUPD (69.9 ± 6.4), compared to DEL (60.8 ± 8.6; *P* < .01). For the verbal subtests, individuals with an mUPD had higher scores for "information" (*P* < .05), "arithmetic" (*P* < .05), "vocabulary" (*P* < .01) and "comprehension" (*P* < .01) compared to DEL. There were no differences in PIQ between the two groups, except for the subtest "object assembly" with higher scores for DEL (*P* < .05). The difference between VIQ and PIQ within the individuals differed between individuals with an mUPD and DEL, with a higher VIQ-PIQ difference for mUPD (*P* < .001). Individuals with an mUPD were more likely to have VIQ exceeding PIQ (OR = 31.6). There were no differences in FSIQ and academic achievement between the two groups.	No separate analyses for children and adults.<br><br>Probably partly the same study population as Joseph et al (2001), Butler et al (2004), Hartley et al (2005), Zarcone et al (2007), Dykens et al (2008), Holsen et al (2009) and Honea et al (2012).
		Health problems, metabolic parameters, body composition, GH secretion and deaths	
Coupaye et al (2016)	Presence of scoliosis, hypogonadism, hypothyroidism, DM; metabolic parameters (fasting glycaemia, HbA1c, fasting insulin, HOMA-IR, lipids, liver enzymes); BMI; body composition (FM, LBM); adipocyte size; REE; hyperphagia[s]; fasting total ghrelin	Hypothyroidism was more frequent among individuals with a DEL, compared to mUPD (37% vs. 11%; *P* = .01). The frequency of scoliosis, hypogonadism and DM did not differ between the two subtypes. Of the metabolic parameters, only HbA1c levels differed between individuals with a DEL (mean 5.6 ± 0.3%) and mUPD (mean 5.3 ± 0.4%; *P* = .02). BMI was higher in individuals with a DEL (mean 40.9 ± 11.15 kg/m^2) than in individuals with an mUPD (mean 34.6 ± 9.6 kg/m^2; *P* = .02). Both FM and LBM were higher in individuals with a DEL (mean LBM 46.5 ± 11.6 kg; mean FM 49.0 ± 18.4 kg), compared to mUPD (mean LBM 39.3 ± 9.7 kg; mean FM 39.7 ± 14.3 kg; *P* = .01 for LBM; *P* = .04 for FM). There were no differences in adipocyte size, REE, hyperphagia score and fasting total ghrelin between the genetic subtypes.	Hypogonadism was present in nearly all subjects (DEL: 98%; mUPD: 92%).<br><br>Probably partly the same study population as Laurier et al (2015), Jauregi et al (2013) and Copet et al (2010).
Laurier et al (2015)	Presence of DM, hypothyroidism,	Individuals with a DEL had a higher BMI (44.0 ± 11.3 kg/m^2 vs. 38.9 ± 10.1 kg/m^2 ; *P* ≤ .01) and were more often diagnosed with sleep apnea or	Probably partly the same study population as Coupaye et al (2016),

	hypertension, hyperlipidemia, sleep apnea/hypoventilation, respiratory problems, scoliosis, skin picking, edema, urinary incontinence, constipation and epilepsy; BMI; medication use.	hypoventilation (42.0% vs. 23.8%; *P* ≤ .05) compared to individuals without a DEL. Individuals without a DEL more often used insulin (19.0% vs. 4.0%; *P* ≤ .05) and psychotropic medication (71.4% vs. 52.5%; *P* ≤ .05) than individuals with a DEL. Presence of DM, hypothyroidism, hypertension, hyperlipidemia, respiratory problems, scoliosis, skin picking, edema, urinary incontinence, constipation and epilepsy did not differ between the two groups.	Jauregi et al (2013) and Copet et al (2010).
Sinnema et al (2011c)	Presence of cardiovascular, respiratory, gastro-intestinal, genitourinary, endocrine, neurologic, orthopedic, dermatologic, ophthalmologic and otolaryngologic disease; predicted 1-year mortality	Pneumonia (11% vs. 2%; *P* = .05), excessive daytime sleepiness (82% vs. 64%; *P* = .05), disturbed sleep (30% vs. 11%; *P* = .02), anemia (11% vs. 2%; *P* = .04) and urinary incontinence (25% vs. 4%; *P* < .01) were significantly more frequent among individuals with an mUPD compared to DEL. Osteoporosis (24% vs. 5%; *P* = .02), knee problems (11% vs. 0%; *P* = .02), edema (67% vs. 43%; *P* = .02) and ear problems (24% vs. 5%; *P* < .01) were significantly more frequent among individuals with a DEL compared to mUPD. The predicted 1-year mortality was higher for individuals with an mUPD (mean Charlson index: 0.66) than for individuals with a DEL (mean Charlson index: 0.20; *P* = .03). There were no differences in cardiovascular, neurologic and ophthalmologic disease between the genetic subtypes.	Probably partly the same study population as Sinnema et al (2011a, 2011b), Maas et al (2010) and as the current manuscript.
Grugni et al (2011)	GH secretion (GHRH arginine test)	Mean [SE] peak GH response and integrated GH secretion were lower in individuals with an mUPD (peak: 4.6 [1.6]; integrated: 228.3 [71.6]) than in individuals with a DEL (peak: 9.1 [1.8]; integrated: 514.9 [127.6]; *P* < 0.005 for all). There were no differences between DEL-1 and DEL-2. IGF-I levels did not differ between the genetic subtypes.	N/A
Smith et al (2003)	Deaths	Nine individuals with a known genotype died, of which 5 with a DEL (56%) and 4 with an mUPD (44%). Compared to the living individuals with a known genotype (17 DEL [77%], 4 mUPD [18%]), relatively more individuals with mUPD had died (50% vs. 23%; *P* < .05). Cause of death did not differ between the two groups.	N/A

Abbreviations: ABC-J, Aberrant Behavior Checklist Japanese version; BMC, bone mineral content; BMD, bone mineral density; BMI, body mass index; CBC, Compulsive Behavior Checklist; CBCL, Child Behavior Checklist; CSF, cerebrospinal fluid volume; DBC-A, Developmental Behavior Checklist for Adults; DM, diabetes mellitus; ERP, event related potential; ESS, Epworth Sleepiness Scale; FM, fat mass; fMRI, functional magnetic resonance imaging; FRPQ, Food Related Problem Questionnaire; FSIQ, full scale intelligence quotient; GH, growth hormone; GHRH, growth hormone releasing hormone; GMV, gray matter volume; HbA1c, glycated hemoglobin; HOMA-IR, Homeostatic Model Assessment for Insulin Resistance; HQ, Hyperphagia Questionnaire; ID, intellectual disability; IQ, intelligence quotient; LBM, lean body mass; MVS, matching (Crichton or Mill Hill) vocabulary scale; OPCRIT, Operational Criteria Checklist for psychotic and affective illness; PARS, Pervasive Developmental Disorders Autism

Society Japan Rating Scale; PAS-ADD, Psychiatric Assessment Schedule for Adults with Developmental Disabilities; PDD, pervasive developmental disorder; PDD-MRScale, pervasive developmental disorder mental retardation scale; PWS, Prader-Willi syndrome; RBQ, Repetitive Behavior Questionnaire; RCM, Raven's Coloured Matrices; REE, respiratory energy expenditure; RR, risk ratio; RSPM, Raven's Standard Progressive Matrices; SA-SDQ, Sleep Apnea subscale of the Sleep Disorders Questionnaire; SIB, Scales of Independent Behavior; SSRI, selective serotonin reuptake inhibitors; TFEQ, Three-Factor Eating Questionnaire; TICV, total-intracranial volume; VABS, Vineland Adaptive Behavior Scales-II; VMI, visual motor integration; VMIS, Visual Motor Integrations Scale; WAIS-III, Wechsler Adult Intelligence Scale III; WAIS-R, Wechsler Adult Intelligence Scale – Revised; WIS, Wechsler Intelligence Scale; WISC-III, Wechsler Intelligence Scale for Children-III; WJB, Woodcock-Johnson Psychoeducational Battery; WJB-R, Woodcock-Johnson Psychoeducational Battery – Revised; WMV, white matter volume; Y-BOCS, Yale-Brown Obsessive Compulsive Scale

[a] The Y-BOCS is a semi-structured interview and was used to assess the severity of the compulsive symptoms, based on five items: time spent doing compulsions, degree of distress due to compulsions, degree of interference in social activities, and ability to resist and control compulsions. [b] The CBC is designed for individuals with an intellectual disability to assess the presence of five groups of compulsive behavior: completeness, cleaning, ordering, checking and touching, and deviant grooming. [c] The RBQ was used to assess the occurrence of repetitive behaviors, grouped in five categories: stereotype behavior, compulsive behavior, restricted preferences, insistence on sameness, and repetitive speech. Answers on all questionnaires were provided by caregivers. [d] The primary psychiatric diagnoses were "any psychotic features", "bipolar disorder (nonpsychotic)", "anxiety disorder", "major depressive disorder", intermittent explosive disorder", and "excoriation (skin picking) disorder". [e] The ABC-J was used to assess the extent of problem behaviors on five subscales: irritability and agitation, lethargy and social withdrawal, stereotypic behavior, hyperactivity and noncompliance, and inappropriate speech. The FRPQ is an informant-based questionnaire to assess food-related behavior on three subscales: preoccupation with food, impairment of satiety, and other food-related negative behaviors. The PARS was used to evaluate current autistic states and consists of five subscales: interpersonal skills, communication, obsession, problematic behaviors, and hypersensitivity. [f] The DBC-A is an assessment instrument to assess behavioral and emotional disturbance on six subscales: disruptive, self-absorbed, communication disturbance, anxiety/antisocial, social relating, depressive. [g] The HQ was used to assess (the severity of) food-related preoccupations and problems on three subscores: behavior, drive, severity. [h] The TFEQ assess the degree of dietary restriction, eating disinhibition, and hunger level. [i] The PAS-ADD is a semi-structured interview schedule for assessing psychopathology in individuals with an ID. [j] The ESS measures the general level of daytime sleepiness. The SA-SDQ measures the presence of sleep apnea. [k] The WAIS-III was used to measure IQ (FSIQ, VIQ, PIQ) and fourteen subtests ("digit span", "information", "vocabulary", "arithmetic", "comprehension", "similarities", "letter-number-sequencing", "picture completion", "picture arrangement", "block design", "object assembly", "digit symbol coding", "matrix reasoning", "symbol search"). [l] The OPCRIT is a checklist for psychopathology. [m] The CBCL was used to assess behavioral disturbances on internalizing and externalizing domains. [n] The VABS was used to assess the performance of behaviors required for person or social self-sufficiency. [o] The WJB(-R) is a battery to measure general intellectual ability, specific cognitive abilities, scholastic aptitude, oral language, and academic achievement. [p] The PDD-MRScale was used to screen for the spectrum of PDD on three aspects: social behavior, communication, stereotyped behavior. [q] The CPT-AX is a Go – No Go task to evaluate inhibitory control. [r] The Reiss Screen was used to assess maladaptive behavior on eight scales (aggressive behavior, autism, psychosis, paranoia, avoidant, behavioral depression, physical depression, dependent personality disorder) and six single items (drug/alcohol abuse, overactive, self-injury, sexual problem, suicidal tendencies, stealing). [s] Hyperphagia was assessed with the Dykens Hyperphagia Questionnaire which measures the (severity of) food-related preoccupations and problems in PWS. The questionnaire has tree subscores: hyperphagic behavior, hyperphagic drive, hyperphagic severity.
